# Towards a high-density photonic tensor core enabled by intensity-modulated microrings and photonic wire bonding

**DOI:** 10.1038/s41598-023-27724-y

**Published:** 2023-01-23

**Authors:** Enxiao Luan, Shangxuan Yu, Mahsa Salmani, Mohammadreza Sanadgol Nezami, Bhavin J. Shastri, Lukas Chrostowski, Armaghan Eshaghi

**Affiliations:** 1 Huawei Technologies Canada Co., Ltd. 19 Allstate Parkway, Markham, Ontario L3R 5A4 Canada; 2 Department of Electrical and Computer Engineering, The University of British Columbia, 2332 Main Mall, Vancouver, British Columbia V6T 1Z4 Canada; 3 Department of Physics, Engineering Physics & Astronomy, Queen’s University, Kingston, Ontario KL7 3N6 Canada

**Keywords:** Engineering, Mathematics and computing, Optics and photonics

## Abstract

We propose a photonic processing unit for high-density analog computation using intensity-modulation-based microring modulators (IM-MRMs). The output signal at the fixed resonance wavelength is directly intensity modulated by changing the extinction ratio (ER) of the IM-MRM . Thanks to the intensity-modulated approach, the proposed photonic processing unit is less sensitive to the inter-channel crosstalk. Simulation results reveal that the proposed design offers a maximum of 17-fold increase in wavelength channel density compared to its wavelength-modulated counterpart. Therefore, a photonic tensor core of size 512 $\times$ 512 can be realized by current foundry lines. A convolutional neural network (CNN) simulator with 6-bit precision is built for handwritten digit recognition task using the proposed modulator. Simulation results show an overall accuracy of 96.76%, when the wavelength channel spacing suffers a 3-dB power penalty. To experimentally validate the system, 1000 dot product operations are carried out with a 4-bit signed system on a co-packaged photonic chip, where optical and electrical I/Os are realized using photonic and electrical wire bonding techniques. Study of the measurement results show a mean squared error (MSE) of 3.09$\times$10$^{-3}$ for dot product calculations. The proposed IM-MRM, therefore, renders the crosstalk issue tractable and provides a solution for the development of large-scale optical information processing systems with multiple wavelengths.

## Introduction

Computational requirements and energy expenditures have escalated rapidly, to either process the exponentially increased data generated by ultra-high-speed mobile networks or to address the demand for accelerating artificial intelligence ^1^. However, current state-of-art electronic processors, which have developed with startlingly rapid progress in the past decades, are approaching their growth limit subject to Moore’s Law. It can be foreseen that if progress continues along the current route, these computational requirements will rapidly become prohibitive technically and economically ^2^. Photonic platforms have been regarded as ideal candidates for analog processing of optical communication signals, providing a framework for a new class of information processing machines ^3^. Compared to electrical counterparts, photonic circuits have their predominant advantages: optical signals traveling at the speed of light can be manipulated by transmission modulation, experience lower attenuation and generate less heat as a function of distance ^3^. Many application-specific optical processors have been exploited for addressing mathematical ^4,5^ and signal processing ^6,7^ tasks with performance enhanced by orders of magnitude.

Integrated photonics has drawn tremendous attention due to its capability to generate, manipulate, and detect optical signals on a single chip. Leveraging photonic integrated circuits (PICs) manufactured using CMOS compatible processes, one can build up miniaturized photonic processing systems with high yield and low cost. According to the requirements of the light source, photonic processing systems can be divided into two categories, coherent architectures and multiwavelength architectures. For the coherent architecture, the coherent input light is employed in an array of beam splitters and phase shifters to perform matrix transforms using interference between different paths ^3^. The Mach-Zehnder interferometer-based (MZI) mesh is the dominant linear photonic processing network with coherent input signals. It is a well-studied and mature architecture for matrix multiplications in computational systems, including applications in optical neural networks ^8,9^, quantum transport simulations ^10^, reconfigurable optical delay lines ^11^ and singular value decomposition ^12^. However, coherent optical interconnects exhibit sensitivity to the optical phase, which requires calibration after each MZI mesh layer ^13^. In addition, since coherent architectures require a single optical phase reference, only a single source laser can be employed. This required the laser to generate high optical power sufficient for the entire system. In contrast to coherent systems, multiwavelength architectures use incoherent signals generated by individual light sources at different wavelengths or a single source that produces multiple wavelengths to carry and process the information. Leveraging wavelength-division multiplexing (WDM), each input signal is an analog optical power at a given wavelength processed in parallel by a bank of modulators.

An integrated multiwavelength architecture, namely broadcast-and-weight, first proposed ^14^ and demonstrated ^15^ using cascaded microring resonators (MRRs) in the silicon photonic platforms, has proven its unique capabilities in information processing applications including in photonic neural networks ^16^, wireless signal processing  ^17^$$^{,}$$ ^18^, and nonlinear programming ^19^. Incoming signals assigned to different wavelength carriers are wavelength division multiplexed and weighted by a photonic weight bank, that is realized by tuning MRR modulators. The signals are then summed up by full power differential detection. Typically, MRR modulators are wavelength-modulated. The resonant peak of the MRR shifts when the refractive index of the waveguide core changes through the thermo-optic effect or the plasma dispersion effect. This results in transmission intensity variation at the carrier wavelength. One of the drawbacks of this modulation technique, particularly in a large-scale system ^20^, is the inter-channel crosstalk issue. Due to this issue, the optical modulation amplitude of the individual wavelength channels needs to be minimized, which, as a result, limits the scalability of the photonic processing system. Another multiwavelength architecture for photonic computing has been demonstrated recently by utilizing phase-change-material (PCM) memory crossbar arrays, which enables a speed of trillions of multiply-accumulate (MAC) operations per second  ^21^. However, in this approach, signals are inscribed based on the absorption rate of the PCM patch on the waveguide. Part of the input light power is dissipated by the absorption. In addition, signals carried at different wavelengths are combined on the bus waveguide through directional couplers. These couplers will generate additional losses due to the broadband coupling. Although no inter-channel crosstalk is present, the crossbar system shows lower energy efficiency compared to the MRR-based system.

**Figure 1:**
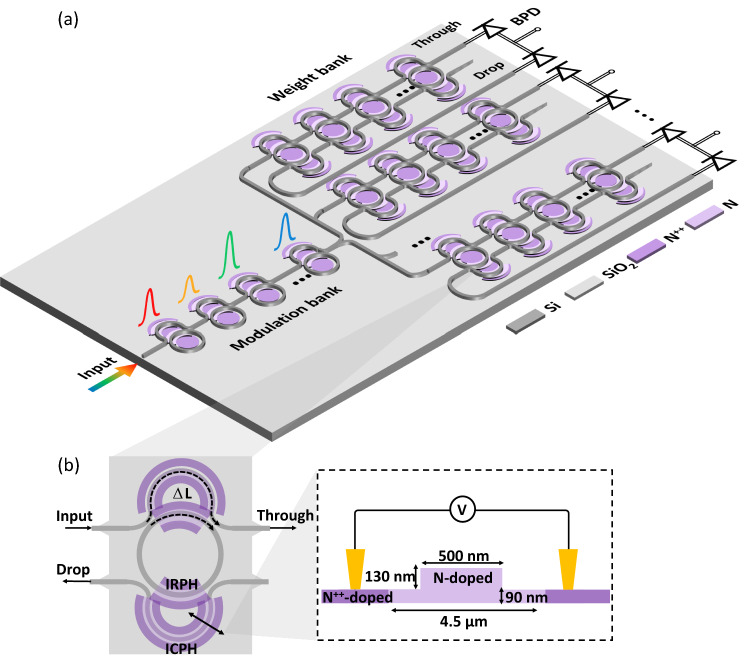
(**a**) Schematic of the proposed photonic processing system using IM-MRMs. For intensity modulation, WDM input light passes through two groups of IM-MRM arrays, as the multiplier set (modulation bank) and multiplicand set (weight bank), respectively. The modulated light at different wavelengths is then accumulated by the BPD at the readout. (**b**) Schematic of one add-drop IM-MRR filter and the cross-section of the doped waveguide, which forms ICPHs and IRPHs in the coupling region and the resonator region, respectively.

This paper investigates a fixed-wavelength intensity modulation scheme for a WDM-based photonic signal processing system. As shown in Figure 1a, interferometric coupling-based microring modulators (MRMs), which contain index-modulation components (shown in purple) in the coupling region and the resonator region, are introduced. Incoming WDM sources are first modulated by the all-pass MRMs in the modulation bank, and then weighted by the add-drop MRR filter arrays in the weight bank at each wavelength. By adjusting the coupling strength and the resonance condition, direct intensity modulation can be achieved at their fixed resonance wavelengths with negligible inter-channel crosstalk. At last, balanced-photodetectors (BPDs) are employed at the output for multiwavelength intensity summation. Simulations results show that when aiming for a 3-dB power penalty, IM-MRMs can improve the wavelength channel spacing by a factor of seventeen compared to the wavelength-modulated MRMs. Using photonic wire bonding (PWB) technique, the photonic chip that includes our proposed system based on IM-MRMs is assembled on a printed circuit board (PCB) and dot product calculation is demonstrated. PWB involves three-dimensional photosensitive polymer-based waveguide writing between different platforms after the die placement, which relaxes the requirement for fine alignment ^3^. Based on the experiments done using the packaged photonic chip, a mean squared error of 3.09 $\times$ 10$^{-3}$ is observed for 1000 multiplications with random input values. Moreover, to study the performance of the proposed scheme in a large-scale system, a convolutional neural network simulator is built using a co-simulation environment in Lumerical and Python. Simulations based on MNIST handwritten digit recognition dataset shows that the architecture can achieve an accuracy of 96.76%. The measurement and simulation results show that the proposed IM-MRMs can be the building block of the large-scale optical information processing systems which do not suffer from the inter-channel crosstalk.

## Intensity-modulated microring modulators

### Design

Interferometric coupling-based (or two-point coupled MZI-based) MRRs have been utilized for hitless-switching ^22^, high-speed modulation ^23^, PAM-4 modulation ^24^, photonic delay line ^25^, single-photon sources ^26^, and post-fabrication correction ^27^. Recently, a wideband tunable add-drop multiplexer is reported using interferometeric coupling-based modulators ^28^. The proposed design utilizes the similar intensity modulation scheme to achieve ’connection’ and ’disconnection’ of filter channels. However, this multiplexer needs 4 directly-coupled MRRs as the Vernier filter, and requires an additional photodetector for tuning and calibration, which inhibits its large-scale application at multiwavelengths.

The schematic in Figure 1b illustrates the proposed IM-MRM used in our high-density photonic processing system. By adding an unbalanced MZI acting as a tunable coupler, the effective coupling ratio changes when the sinusoidal spectral response of the MZI coupler shifts in wavelength ^22^. Accordingly, the extinction ratio of the resonant peak can be modulated in the MRM. The length difference $\Delta L$ between the unbalanced MZI coupler arms (as shown in Figure 1b) determines the free spectral range (FSR) of the device, according to the following equations:

1 $$\begin{aligned} FSR_\text {MZI} = \frac{FSR_\text {MRR}}{m}, \end{aligned}$$

2 $$\begin{aligned} m=\frac{\Delta L}{2\pi R}, \end{aligned}$$

where, $FSR_\text {MZI}$ and $FSR_\text {MRR}$ represent the FSR of the MZI coupler and the MRR, respectively, and *R* is the radius of the MRR. According to different non-negative values of *m*, the coupling spectrum and the ring resonance behave as either co-period or periodic resonance suppressed ^22^. To realize intensity modulation at a fixed wavelength, $\Delta L$ is set to be $\pi R$ (*m* = 1/2) to accommodate the phase shifting mechanism in the MZI arm. A device for larger values of *m* can be designed by sacrificing the footprint to improve the tuning efficiency. Analytical equations used towards the design of the IM-MRM can be found in Section S1 of the Supplementary Information.

The cross-section in Figure 1b shows a doped silicon waveguide, namely the photoconductive heater, to serve as the index-modulation and resonant peak-tracking component. N-doped resistive heaters integrated into MRRs have shown photoconductive effects with high responsivities for automatically tuning and stabilizing the filter’s resonant peak to the wavelength of interest ^29^. Unlike germanium (Ge) deposition ^30^ or PIN diode implantation ^31^, N-doped resistive heaters do not require dedicated defect implantation steps, additional material depositions, dedicated photodetectors, or optical power taps. This allows for a low-cost, compact and straightforward method for modulating large-scale MRR systems. Two types of N-doped photoconductive heaters are involved in our IM-MRM design. An in-coupler photoconductive heater (ICPH) serves as the effective coupling ratio modulator in the MZI coupler, and an in-resonator photoconductive heater (IRPH) serves as the resonant peak monitor and wavelength compensator in the MRR (shown in Figure 1b). Although N-doped photoconductive heaters have been employed in optical computing ^16^, biosensing ^32^ and data transmission ^29^ systems, to our knowledge, this is the first demonstration of doped silicon-based multiwavelength optical information processing systems leveraging intensity modulation at a fixed wavelength.

### Characterization and control

**Figure 2:**
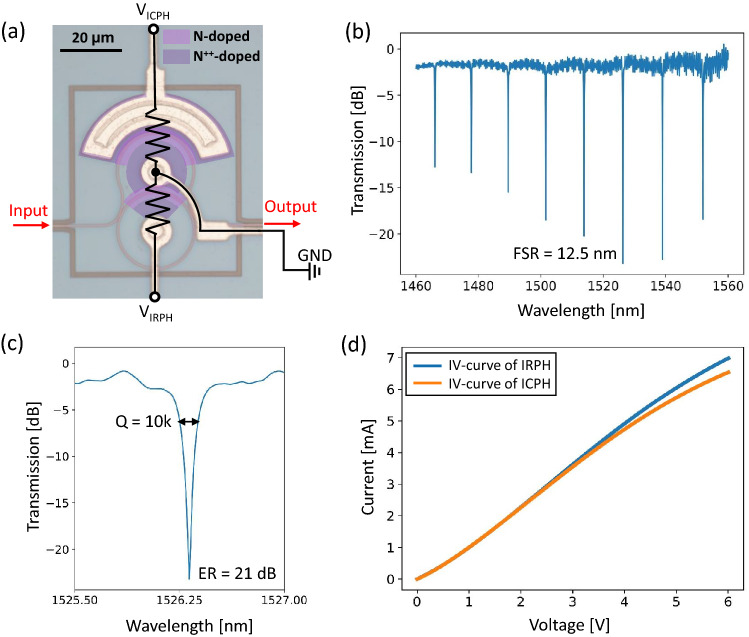
(**a**) Microscopic image of the all-pass IM-MRM design overlaid with a circuit description of the integrated ICPH and IRPH. (**b**) Measured transmission spectrum of the fabricated IM-MRM after normalization, showing a doubled FSR. (**c**) A closer look at the resonant peak at 1526.25 nm. (**d**) Measured IV curves for integrated ICPH and IRPH with voltages varying from 0 to 6 V.

All the devices described in this paper were designed using the open-source layout editor KLayout and SiEPIC-Tools, and fabricated on a silicon-on-insulator (SOI) wafer with 220-nm-thick silicon and 2-$\upmu$m-thick buried oxide layers through a SiEPICfab Multi-Project Wafer fabrication run by Applied Nanotools Inc. Details of the optical characterization setup can be found in Section S2 of the Supplementary Information. Figure 2a shows a microscopic image of the proposed all-pass IM-MRM with integrated ICPH and IRPH components, which share the ground for space-saving consideration. N-doped and N$^{++}$-doped regions are false-colored. It is worth noting that due to the compact footprint (*m* = 1/2) and a shared ground, undesired effects caused by thermal and electrical crosstalk are observed between the ICPH and IRPH, which needs to be optimized in future designs (discussed in “Discussion and conclusion” Section). The radius is 15 $\upmu$m, and the coupling gap is 200 nm. The transmission spectrum of the fabricated IM-MRM, after calibration for removing the insertion loss of the grating couplers, is shown in Figure 2b. The FSR is measured at 12.5 nm, which is approximately doubled compared with a point-coupling-based MRM with the same radius. This matches the design above with $\Delta L = \pi R$, considering a group index of 3.85 for the N-doped waveguide. The noise in the transmission spectrum is because of the high return loss of the grating couplers suffering from high levels of back reflection in the system, which can be improved with optimization. A zoomed-in plot aiming for one resonant peak at 1526.25 nm is depicted in Figure 2c, indicating a quality factor (*Q*-factor) of $\sim$10,000 and an ER of 21 dB. In Figure 2d, IV curves for ICPH and IRPH are presented when no light is incident (dark current).

**Figure 3:**
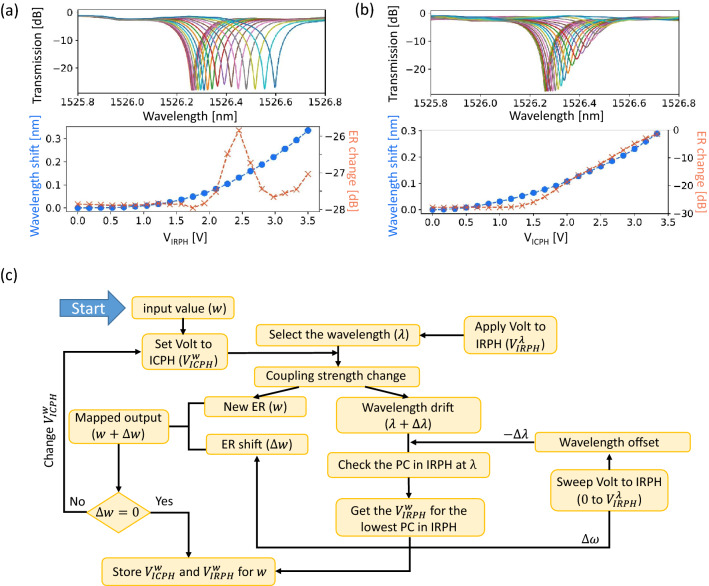
(**a**) Measured transmission spectra of the proposed IM-MRM after normalization when a voltage is applied to the IRPH from 0 to 3.5 V, as well as the extracted wavelength shift and ER change (**b**) Measured transmission spectra of the proposed IM-MRM after normalization when a voltage is applied to the ICPH from 0 to 3.5 V, as well as the extracted wavelength shift and ER change. (**c**) Flow diagram of the intensity-modulation algorithm. PC: photocurrent.

The resonance wavelength and ER of the IM-MRM can be manipulated by applying voltages across the IRPH ($V_\text {IRPH}$) and ICPH ($V_\text {ICPH}$) ^33^. As shown in Figure 3a, when only $V_\text {IRPH}$ is applied (from 0 to 3.5 V), the resonant peak shows a 350 pm red-shift, and the ER remains at about 27 dB. While, when only applying $V_\text {ICPH}$, the resonant peak shifts and changes the ER from 27 dB to 1.25 dB (Figure 3b). Therefore, by adjusting both $V_\text {IRPH}$ and $V_\text {ICPH}$ and changing the ER of the resonant peak, we could realize an intensity modulation scheme, while the peak position is maintained without any wavelength shift. The flow diagram of the intensity-modulation algorithm is outlined in Figure 3c. First, a wavelength of interest ($\lambda$) is selected, which is achieved by applying a voltage to the IRPH ($V_\text {IRPH}^{\lambda }$). For information encoding, a “discrete analog” encoding/decoding scheme is employed to implement direct value mapping for translating the digital number to an analog value ^34,35^. More details about the “discrete analog” scheme can be found in “Dot product engine” Section. Here, we apply a voltage to the ICPH ($V_\text {ICPH}^{w}$) to achieve a transmission at the resonant peak for representing the input values (*w*) according to the measured ER from Figure 3b. This causes a drift in the resonant peak to another wavelength $\lambda + \Delta \lambda$. To compensate for the unwanted drift, a wavelength offset scheme is employed using the photodetection mechanism of the IRPH. The voltage applied to the IRPH is swept from 0 to $V_\text {IRPH}^{\lambda }$ while $V_\text {ICPH}^{w}$ is kept fixed. the photocurrent generated in the IRPH at wavelength $\lambda$ is monitored to find the new resonant wavelength ($V_\text {IRPH}^{w}$). It is worth noting that changing the voltage applied to the IRPH offsets the wavelength shift and changes the ER simultaneously, although at much lower efficiency ($\Delta w<$ *w*). A calibration algorithm is used to tune $V_\text {ICPH}^{w}$ and minimize $\Delta w$. At last, the calibrated $V_\text {ICPH}^{w}$ and $V_\text {IRPH}^{w}$ voltage pair are stored for encoding the input value, *w*.

**Figure 4:**
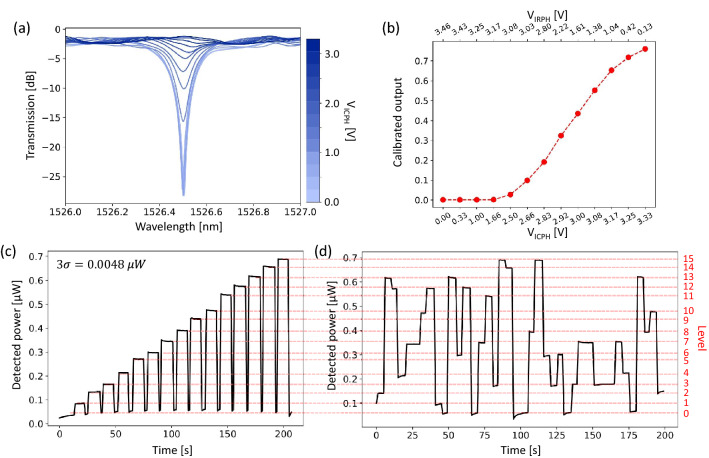
(**a**) Measured transmission spectra after normalization with different $V_\text {ICPH}$ and $V_\text {IRPH}$ voltage pairs for intensity modulation operations at 1526.5 nm. (**b**) Calibrated outputs of the IM-MRM at the resonant peak. (**c**) 16 distinguishable power levels in consecutive ascending order with 3$\sigma$ = 0.0048 $\upmu$W. (**d**) Arbitrary levels reached independently among 16 intermediate levels.

Normalized transmission spectra with discrete ER values of the resonant peak fixed at 1526.5 nm are depicted in Fig. 4a. By applying the voltage to the ICPH from 0 to 3.3 V followed by changing the corresponding voltage on the IRPH (obtained through the intensity-modulation algorithm), the ER of the resonant peak varies from -27 to -1.25 dB with no apparent wavelength shift. Figure 4b shows the calibrated output extracted from resonant peaks at 1526.5 nm in Fig. 4a with different $V_\text {ICPH}$ and $V_\text {IRPH}$ voltage pairs. It can be observed that when $V_\text {ICPH}$ is larger than 1.66 V, the transmitted light starts to increase because of the change of the coupling strength, and $V_\text {IRPH}$ drops from 3.46 to 0.13 V accordingly for wavelength offsetting. Figure 4c demonstrates the multi-level operation with 4-bit distinct output levels. The transmission returns to level 0 for erasure ($V_\text {ICPH}$ = 0 V and $V_\text {IRPH}$ = 3.46 V) between each level. Since the applied voltage and the transmitted output show a nonlinear relationship during the intensity modulation operation, a simple predistortion step is introduced by applying a series of voltage pairs with unequal increments to realize approximately linear output levels with an interval of $\sim$0.05 $\upmu$W in Fig. 4c. More precise linear distributions can be achieved by finely adjusting the voltage pairs for each power level. Three standard deviations (3$\sigma$) of 0.0048 $\upmu$W is observed in Fig. 4c (SNR = 10.4 dB); therefore 7.2-bit precision could be achieved. It has been reported that IRPH-based MRMs allow continuous, multichannel control of the weight bank with accuracy up to 8.5 bits ^36^. By employing a “dithering” control scheme to monitor and stabilize the entire optical link, environmental changes in the system can be compensated with high precision ^36^. By selecting predistorted voltage pairs applied to ICPH and IRPH, one can reliably move the transmitted power between these known intermediate levels with high repeatability (shown in Fig. 4(d) with a sampling rate of 1 Hz). Detailed information on the predistortion step can be found in Section S2 of the Supplementary Information.

**Figure 5:**
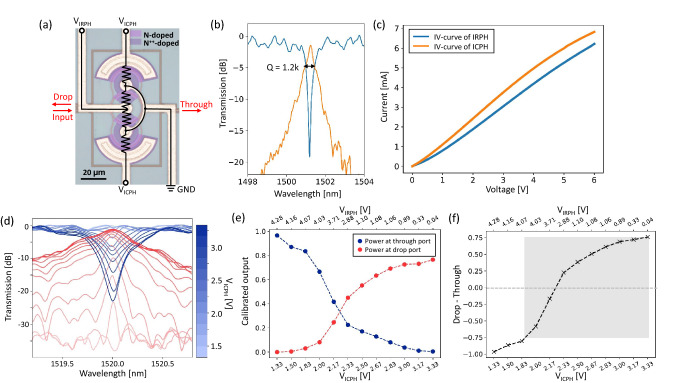
(**a**) Microscopic image of the add-drop IM-MRR weight filter design overlaid with a circuit description of the integrated ICPH and IRPH. (**b**) Measured normalized transmission spectra at through (blue) and drop (orange) ports. (**c**) Measured IV curve for one of the ICPH and IRPH voltage pairs changing from 0 to 6 V. (**d**) Measured normalized transmission at through (blue) and drop (red) ports for intensity modulation at 1520 nm. (**e**) Calibrated outputs of the add-drop IM-MRR at the resonant peak. (**f**) Measured weight range from the subtraction between the calibrated output at drop and through ports. The grey region represent the symmetrical range relative to 0.

Similarly, an add-drop IM-MRR filter with a radius of 15 $\upmu$m was also fabricated to serve as the positive and negative signed information encoding element. As shown in Fig 5a, another MZI coupler connecting add and drop ports of the IM-MRR is implemented, and both MZI couplers are designed symmetric to the ring with coupling gaps of 200 nm to reduce the IL ^37^. Figure 5b presents through and drop ports transmission spectra. A reduced* Q*-factor of $\sim$1200 is observed compared to the all-pass one, due to the higher absorption loss in the resonator which contains the IRPH with twice the size. In Fig. 5c, IV curves are measured with no incident light. Normalized transmission spectra at through (blue) and drop (red) ports are measured by applying different $V_\text {ICPH}$ and $V_\text {IRPH}$ voltage pairs and are presented in Fig 5d along with the calibrated output values at resonance wavelength (Fig. 5e) following the procedure in Fig. 3c and  “Characterization and control” Section. Measured weight values for the add-drop IM-MRR are plotted in Fig 5f. By subtracting the calibrated output power between drop and through ports, a weight range from -1 to 0.75 is obtained. The insertion loss of the modulator causes the 0.25-deduction of the maximum value at the positive side. Therefore, to get a symmetrical weight range relative to 0, the range of [-0.75, 0.75] is selected. Since multiplication can be interpreted as one input value being weighted by another, we define the mapping between voltage pairs and the transmitted power as the input mapping for the all-pass IM-MRM, and the weight mapping for the add-drop IM-MRR, respectively, in the rest of this paper.

## Scalability

It is known that the number and the bandwidth of optical signals are restricted by the ability of the modulators to tune each wavelength channel independently ^13^. In WDM systems, two factors determine the maximum wavelength channel density, the FSR and the linewidth of the MRR. Namely, the resonator’s finesse ($\mathscr {F}$ = FSR/linewidth) sets the upper fan-in limit: $N\le \mathscr {F}$, where *N* is the number of wavelength channels. However, unlike demultiplexers, weight banks in the broadcast-and-weight architecture are reconfigurable, requiring independent modulations for every signal over a transmission range ^13^. In addition, weight banks contain two output ports (through and drop). Input signals carried by different wavelengths are proportionally multiplexed and demultiplexed by a series of add-drop MRRs in the weight banks, where the inter-channel crosstalk results from the overlap of the optical pass-bands of the modulators. Therefore, a wider wavelength channel spacing than the linewidth of MRRs is always necessary to minimize the crosstalk impact.

Impacts of crosstalk in MRR-based WDM systems have been numerically investigated, in which the level of crosstalk is specified by the level of isolation between adjacent channels ^38–40^. As for the broadcast-and-weight architecture, we utilize a similar metric to allow for the crosstalk effect to be incorporated into the power budget of the weight banks. The metric is defined as the weighted power range for a signal when it is impaired by crosstalk relative to the range in the absence of crosstalk sources. It has been reported that a 3-dB power penalty caused by the crosstalk is observed in the weight banks tuned by wavelength modulation, when the minimum wavelength channel spacing falls between 3.41 and 4.61 times linewidth ^13^. Considering a finesse of 368 and a minimum wavelength channel spacing of 3.41 times linewidth, up to 108 wavelength channels can be supported in the broadcast-and-weight system ^41^. Finesse of 368 is experimentally demonstrated by a passive MRR with a radius of 1.5 $\upmu$m. However, it will be challenging to achieve with active modulation components ^42^. A more manufacturable MRR radius is 5 $\upmu$m for an active MRM with an acceptable estimated Q-factor of 10,000. Therefore, the resonant peak linewidth is around 153 pm, revealing that only 34 wavelength channels can be supported. As a result, a solution is required to address the scalability limit caused by the inter-channel crosstalk.

**Figure 6:**
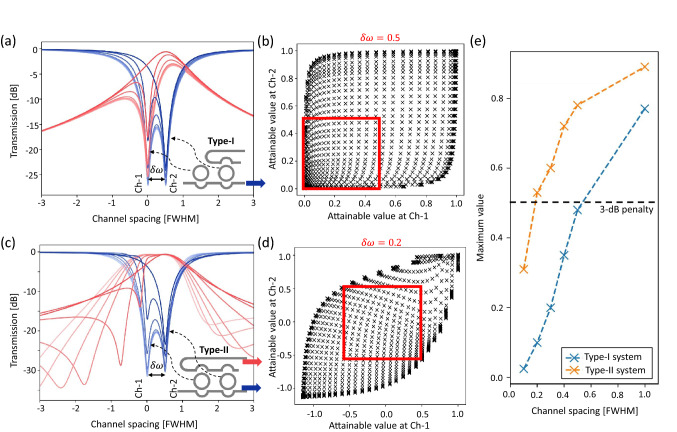
(**a**) Simulated transmission spectra at through (blue) and drop (red) ports of a system with an all-pass MRM cascaded with an add-drop MRR filter (Type-I), the linewidth-normalized channel spacing is set to 0.5. Inset: Schematic of the Type-I system, where the left-hand MRR filter generates the resonant peak at Channel-1 and the right-hand one generates the resonant peak at Channel-2. (**b**) Attainable values for Channel-1 and Channel-2 of Type-I system with $\delta \omega$ = 0.5. The red box shows the usable range of [0, 0.5] for both channels. (**c**) Simulated transmission spectra at through (blue) and drop (red) ports of a system with two cascaded all-pass MRMs (Type-II), the linewidth-normalized channel spacing is set to 0.5. Inset: Schematic of the Type II system, where the left-hand MRR filter generates the resonant peak at Channel-1 and the right-hand one generates the resonant peak at Channel-2. (**d**) Attainable values for Channel-1 and Channel-2 of Type-II system with $\delta \omega$ = 0.2. The red box shows the usable range of [–0.5, 0.5] for both channels. (**e**) Attainable mapping range for two types of IM-MRM cascaded systems with the linewidth-normalized channel spacing varying from 0.1 to 1. The dashed line represents the 3-dB power penalty threshold.

Intensity modulation at a fixed wavelength shows the potential for signal encoding with lower crosstalk penalty and higher wavelength channel density. Since the wavelength is kept fixed through the encoding process, no additional wavelength channel spacing is needed to accommodate wavelength drift. To investigate the scalability of the proposed IM-MRM system, we employed Lumerical’s tools for simulations ^43^. A custom compact model for the IM-MRM was developed in Lumerical INTERCONNECT and used for the crosstalk penalty investigation. Figure. 6(a,c) show the transmission spectra of two types of cascaded IM-MRM systems, and the insets show the schematic of the cascaded rings. The first type is an all-pass MRM cascaded with an add-drop MRR filter (Type-I in Fig. 6a), which is used to investigate the inter-channel crosstalk between modulation banks and weight banks. For the second type, both MRMs are all-pass and cascaded in parallel (Type-II in Fig. 6c). It is used to investigate the inter-channel crosstalk in weight banks only. The wavelength channel spacing is normalized by the linewidth ($\delta \omega$ = $\mathscr {F}/N$ ^13^). This provides an objective comparison between MRM systems with varying linewidths and FSRs. By changing the ER of the resonant peak at Channel-1 (shown as Ch-1 in Fig. 6), the transmission at through (blue) and drop (red) ports at Channel-2 (shown as Ch-2 in Fig. 6) is passively changed due to the crosstalk phenomenon ($\delta \omega$ = 0.5 in both systems). It can be observed that the crosstalk impact in Fig. 6(a,c) are different. In Fig. 6a, the crosstalk penalty at Channel-2 is mainly due to the overlap of the Lorentzian-shaped pass-band at Channel-1, and only the drop port at Channel-2 is strongly affected. While, in Fig. 6c, the inter-channel crosstalk is more complex. Since these add-drop IM-MRMs share two parallel bus waveguides that are parallel-coupled to each MRM, a resonator-like coherent feedback path is created between the resonances of similar wavelengths. The coherent interaction is specifically severe when the resonances are closely spaced. It depends on the bus waveguide’s phase ^13^. The bus-waveguide-induced phase change affects the performance of two adjacent resonant peaks when the modulation method is based on wavelength shifting. For the intensity-modulation-based weight bank system proposed here, we only need to monitor the light intensity at individual resonance wavelengths where the coherent interference has a minimal impact on the amplitude ^44^. Therefore, the transmission at the resonant peak wavelength is only determined by the ER. A detailed derivation of the coherent interference within multiple cascaded MRRs is presented in Section S3 of the Supplementary Information.

The 3-dB power penalty-based value mapping for two types of cascaded MRMs systems are depicted in Fig. 6(b,d) with $\delta \omega$ = 0.5 and 0.2, respectively, both of which present the attainable mapping range (red boxes in Fig. 6(b,d) close to 0.5. Unlike Fig. 6(a,c) which present the transmission spectra obtained by only applying voltage pairs to the IM-MRR at Channel-1, plots in Fig. 6(b,d) are obtained by modulating both IM-MRRs simultaneously. For Type-I systems, only positive values are considered that are obtained from the through port of the system. It requires $\delta \omega$ = 0.5 to allow both MRMs to be independently modulated between 0 and 0.48, as seen in Fig. 6b. For Type-II systems in Figure 6d, a symmetric weight range centred at 0 is investigated that is generated by the differential power between the drop and through ports at each wavelength channel. It allows a smaller wavelength channel spacing ($\delta \omega$ = 0.2) for the same 3-dB power penalty. Figure 6e shows the maximum mapping range as a function of the wavelength channel spacing for both types with $\delta \omega$ varying from 0.1 to 1. Detailed individual mapping plots for each wavelength channel spacing can be found in Section S4 of the Supplementary Information. Considering 3-dB as the tolerable power penalty benchmark, Type-I systems require $\delta \omega>$ 0.5 (blue), while Type-II systems require $\delta \omega$ = 0.2 (orange). Modulation banks can be broadband, such as electro-absorption modulators (EAMs) or electro-optic modulators (EOMs), which do not generate filtered pass-bands in transmission. By implementing broadband modulator as modulation banks, only Type-II crosstalk needs to be considered. The wavelength channel spacing is $\sim$17 times denser than the one from conventional wavelength-modulation-based MRMs (WM-MRMs) ^13,41^ and hence, a tensor core with size of up to 578 can be realized. This demonstrates that the intensity modulation scheme in the MRM-based optical computing system improves the inter-channel crosstalk tolerance, and facilitates using more wavelength channels within one FSR.

It is worth mentioning that the noise due to signal beating may occur at the BPD readout when two resonant peaks are too close such that the difference of the optical frequencies is within the bandwidth of the detector. To eliminate this noise, a frequency filter is needed at the readout to remove the beating frequencies that are larger than the system’s sample rate. For the optical frequencies with a 5 GHz difference at the C-Band, the channel spacing is roughly 40 pm in wavelength. Considering $\delta \omega$ = 0.2 in Type-II system, the FWHM of each resonant peak is $\sim$200 pm. Therefore, to reduce the crosstalk caused by the beating signals with < 5 GHz, the Q-factor of the resonant peak needs to be lower than 7750.

## Dot product engine

### Co-packaging

A dot product computation system that includes a photonic chip with an all-pass IM-MRM and one add-drop IM-MRR weight filter is tested. The photonic chip is co-packaged on a custom PCB, leveraging photonic and electrical wire bonding for optical and electrical inputs/outputs (I/Os). We used a chip-on-board assembly method for co-packaging of the photonic chip. The three main steps for co-packaging are as follows. First, the photonic chip and V-grooves with single-mode fibers are directly mounted on a PCB substrate using UV curable epoxies. In the second step, photonic wire bonding is done using a photonic wire bonder. PWB is a state-of-the-art technique for implementing optical interconnects with polymer waveguides built by in-situ two-photon polymerization ^45,46^, enabling flexible interconnection with a low insertion loss between different material platforms and components (III-V lasers ^47^, SOAs ^48^, optical fibers ^46^ and silicon photonics chips). PWB avoids out-of-plane coupling; thus enabling dense optical I/Os with a pitch down to 25 $\upmu$m ^49^. Furthermore, the PWB technique is fully automatic and has no active alignment requirements, making PWB suitable for mass production ^46^. The third step is electrical wire bonding (EWB). At this step, the aluminum (Al) bond pads on the photonic chip are wire-bonded to the corresponding electroless nickel immersion gold pads on the PCB to implement chip-to-PCB electrical interconnects. An ultrasonic energy is used to attach an Al wire from the photonic chip pads to the PCB pads. We used the wedge-wedge bonder. More information for the co-packaging result can be found in Section S6 of the Supplementary Information.

**Figure 7:**
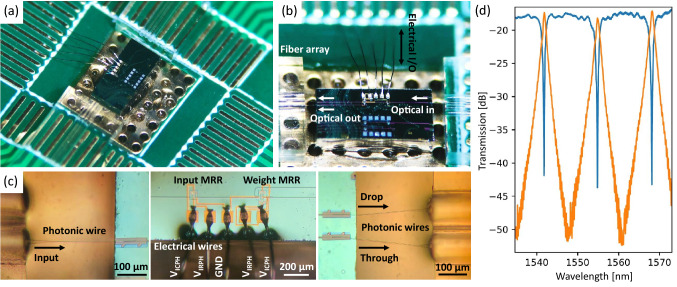
(**a**) Microscopic image of the co-packaged photonic chip on the PCB. (**b**) Zoomed-in image of the mounted photonic chip and V-grooves fiber arrays, showing the optical and electrical I/Os. (**c**) A series of zoomed-in images focusing on the photonic and electrical bonding wires and the photonic chip. (**d**) Measured as-fabricated transmission spectra of the photonic chip at through (blue) and drop (orange) ports.

Figure 7a shows the co-packaged photonic chip on the PCB. The solder under the photonic chip is exposed on the bottom side of the PCB for temperature control. A zoomed-in microscopic image in Fig. 7b presents the mounted photonic chip and V-grooves fiber arrays on the PCB, with optical and electrical I/Os to conduct the signal on and off the photonic chip. A dual-channel optical power meter is employed as the off-chip readout for O/E conversion and power subtraction. Figure 7c presents a series of magnified images focusing on photonic and electrical wire bonds, and the photonic chip. One input MRR (all-pass IM-MRM) for the input encoding and one weight MRR (add-drop IM-MRM) for the signal weighting are cascaded on the chip (Figure S8a), where electrical wires labelled with $V_\text {IRCH}$ and $V_\text {IRPH}$ are used to apply voltage pairs to each MRM. Drive and bias to the packaged photonic chip are provided by the source meter. When no voltage is applied, as-fabricated transmission spectra of the packaged photonic chip measured at through (blue) and drop (orange) ports are depicted in Fig. 7d. An insertion loss around -17.5 dB is observed (-7.5 dB insertion loss per PWB interface considering each IM-MRM has an insertion of -1.25 dB), which might be due to the under etching of the oxide opening at PWB interfaces and their rough sidewalls (see SEM images in Figure S11 of the Supplementary Information).

### Crosstalk characterization

**Figure 8:**
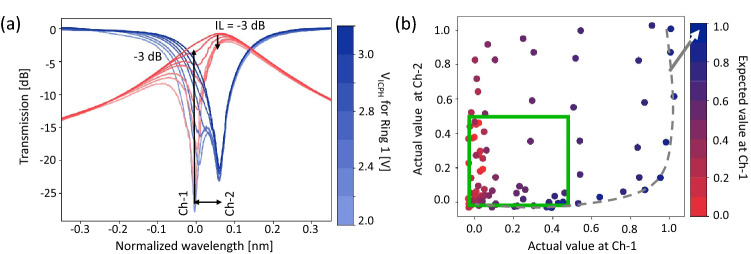
(**a**) Measured transmission spectra at through (blue) and drop (red) ports of Type-I systems, the X-axis represents the wavelength offset from the resonance wavelength of Ch-1. The linewidth-normalized channel spacing is set to 0.5. (**b**) Measured value ranges for Channel-1 and Channel-2. The green box shows the usable mapping range of [0, 0.5]. The colorbar interprets the expected value for Ch-1.

To experimentally evaluate the aforementioned inter-channel crosstalk, different voltage pairs are subsequently applied to both IM-MRMs in the co-packaged chips. Figure 8a shows the normalized transmission spectra at through (blue) and drop (red) ports. Two resonant peaks are observed in the through-port transmission and one in the drop-port transmission. Therefore, the left and right peaks are associated with the all-pass (Channel-1) and the add-drop (Channel-2) IM-MRR filters, respectively. The wavelength channel spacing is set to 0.7 nm ($\delta \omega$ = 0.5) by simply adjusting the $V_\text {IRPH}$ applied to the add-drop IM-MRR filter in Channel-2. By changing the voltage pair of the all-pass IM-MRM, the transmission intensity varies at Channel-1. At the same time, the drop-port transmission at Channel-2 changes accordingly due to crosstalk. As seen in Figure 8a, with different voltage pairs applied to the all-pass IM-MRM, the maximum normalized transmitted power at Channel-1 can reach to -3 dB, while under critical coupling condition at Channel-1, the insertion loss of the add-drop IM-MRR at Channel-2 drops 3 dB. Both observations are consistent with the simulation results in Fig. 6a.

We then applied different voltage pairs to the add-drop IM-MRR filter for generating the attainable mapping values at Channel-2 when different mapping values from 0 to 1 are pre-applied to the all-pass IM-MRM at Channel-1. The mapping value is represented by the detected intensity normalized by the maximum transmission (0 dBm). Figure 8b shows that the measured attainable mapping range for Channel-1 and Channel-2 is limited within [0, 0.5] (green square) due to the inter-channel 3-dB power penalty crosstalk. The variation in the color of dots in the plot represents the expected mapping value pre-applied to the all-pass IM-MRM at Channel-1. However, the measured value at Channel-1 (at the X-axis) is different from the predicted mapping value due to crosstalk. For example, for the dark blue dots (with an expected mapping value of 1.0 at Channel-1), the measured value at Channel-1 will equal 1.0 only when the value at Channel-2 is also set to 1.0. In this scenario, both MRMs represent 1.0 at each channel (no resonant peaks); thus, no inter-channel crosstalk exists. But when the measured value at Channel-2 is not 1.0, the measured value at Channel-1 will drop accordingly, even though the all-pass IM-MRM is consistently pre-applied with 1.0. In conclusion, the experimental data presented in Fig. 8 validates the reliability of the simulation using our custom compact model for IM-MRM and demonstrates that for $\delta \omega$ = 0.5, the power penalty between two adjacent channels is 3 dB.

### Dot product calculation

**Figure 9:**
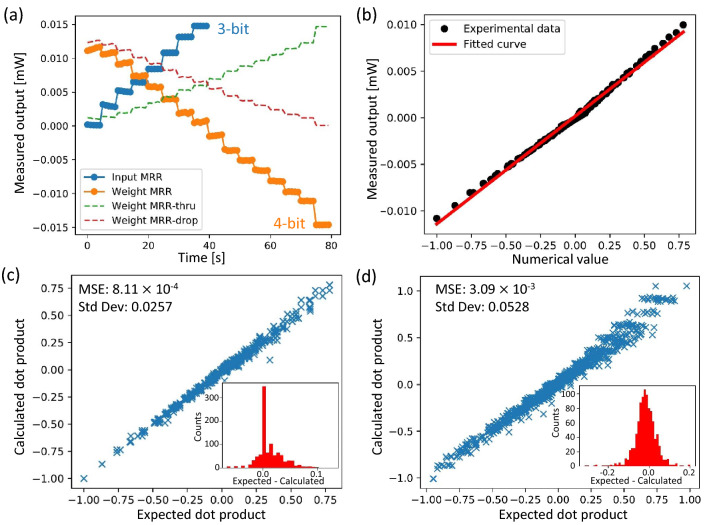
(**a**) Measured power levels of the proposed IM-MRM-based co-packaged chip. The blue steps represent the power level of the all-pass IM-MRM, and the orange steps represent the differential power level of the add-drop IM-MRM after the subtraction between drop and through ports. (**b**) Experimental look-up table for the co-packaged chip using point-to-point mapping scheme with a first-order linear fitting. (**c**) Calculation accuracy for dot product operations with 1000 random digital numbers which are selected from power levels. Inset: The histogram of the Gaussian distribution of the errors. (**d**) Calculation accuracy for dot product operations with 1000 random decimal floating-point numbers rounded to the closest power level. Inset: The histogram of the Gaussian distribution of the errors.

We implement a 4-bit signed weighting system and 3-bit input encoding using the co-packaged chip for our proof-of-concept demonstration of dot product computation. We firstly characterize transmission levels for the all-pass IM-MRM (input MRR in Fig. 7c) and the add-drop IM-MRR filter (weight MRR in Figure 7(c)) with 3-bit and 4-bit precision, respectively, using the aforementioned intensity-modulation algorithm. Both of the IM-MRMs are intensity modulated at the same operational wavelength. Figure 9a illustrates 8 distinguishable power levels for the input MRR (solid blue line) and 16 distinguishable power levels for the weight MRR (solid orange line). The power levels for the weight MRRs are obtained after subtracting each power level between drop (dashed red line) and through (dashed green line) ports. For the input MRR, the power levels are distributed between 0 to 15 $\upmu$W while for the weight MRR, due to the IL, the maximum transmitted power at the drop port can only reach 11.25 $\upmu$W; thus limiting 16 power levels between 11.25 to -15 $\upmu$W. We employ the “discrete analog” scheme to encode inputs and weights to distinct power levels of the input MRR and the weight MRR, respectively ^35^. By normalizing each quantized power level (solid lines in Figure 9a) by the maximum measured output power of 15 $\upmu$W, a point-to-point mapping is realized by endowing voltage pairs with digital information, which can be expressed as:

3 $$\begin{aligned} \begin{aligned} D = \frac{I_\text {volt pair}}{I_\text {max}}, \end{aligned} \end{aligned}$$

where, *D* is the correlated digital number, and $I_\text {volt pair}$ and $I_\text {max}$ are the voltage pair-applied transmission and the maximum transmission of the IM-MRM, respectively. For instance, the voltage pair that allows maximum transmission of the input MRR (15 $\upmu$W) is mapped to 1, and minimum transmission to 0. Therefore, voltage pairs for the 3-bit input MRR can realize the mapping of 8 discrete digital numbers ranging from 0 to 1, while for the 4-bit weight MRR, the 16 digital numbers can be mapped to the range of 0.75 to -1, due to the IL. Figure 9a shows that the power levels were not quantified uniformly as we expected especially for the Weight MRR’s output levels on the negative side, which we believe is due to localized thermal crosstalk and fluctuations. This is consistent with the fact that more applied voltages are required for negative weighting values in Fig. 5f, since more voltages mean more unwanted heat generation. However, the inhomogeneity is fully captured and reproduced in generated digital representations by exploiting the point-to-point mapping scheme. In terms of readout, decoding is required to convert the detected power at the BPD to a digital dot product result. To establish the decoding correlations, a look-up table is needed. In Fig. 9b, an experimental look-up table (128 points) for the co-packaged chip is generated by point-to-point applying digit-endowed voltage pairs to input and weight MRRs according to Figure 9a. The Y-axis shows the differential output power detected by the dual-channel optical power meter. The X-axis shows the dot product result of the mapped digital numbers to the co-packaged chip. The differential output power has an almost linear relationship with respect to dot product results with a first-order linear fit.

Figure. 9(c,d) demonstrate dot product computation using the proposed photonic chip. By randomly encoding digital numbers, selected from power levels in Figure 9a, as the input and the weight to the photonic chip, dot product results are obtained by decoding the readout according using the look-up table and compared with the expected dot product result. The evaluation result of 1000 dot product operations is shown in Figure 9c, with the inset showing a histogram of the error with an MSE of 8.11 $\times$ 10$^{-4}$. Since we only selected existing power levels from two IM-MRMs as inputs and weights, applied voltage pairs are precise; thus, computing errors are mainly from the system fluctuation. Then, dot product computation with random decimal floating-point number is also performed using the proposed photonic chip. Two random decimal floating-point numbers ranging within [0, 1] and [-1, 1] are firstly generated and rounded to the nearest digital number according to power levels in Fig. 9a, then encoded into the system using voltage pairs for the closest digital number. As for decoding, the fitted curve in the look-up table is employed. Figure 9d shows the evaluation result of 1000 dot product operations with decimal floating-point numbers. The inset shows a histogram of the error with an MSE of 3.09 $\times$ 10$^{-3}$. Additional rounding errors and fitting errors can explain the increased MSE during encoding and decoding processes, which can be reduced by improving the bit-precision of the system.

## Performing convolution

We develop a CNN simulator for MNIST handwritten digit recognition, following the DEAP-CNN system described in Ref. ^41^, so as to evaluate the computational performance of the proposed IM-MRM system with limited channel spacing and also to compare the performance of different signal modulation schemes (IM-MRMs vs. WM-MRMs). The CNN starts with two convolutional layers, each of which with 8 kernels of size 3 $\times$ 3 with Rectified Linear Unit (ReLU) non-linear activation function. The convolutional layers are followed by an average pooling layer and, finally, the last two layers of the network are fully-connected layers.

**Figure 10:**
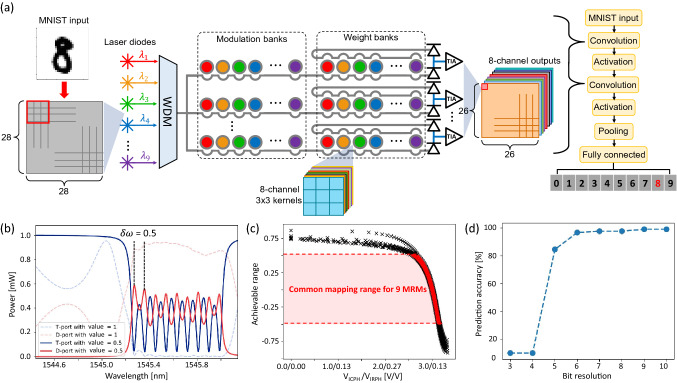
(**a**) Schematic of the proposed CNN architecture using IM-MRMs for modulation and weight banks. The kernel dimension is 3 $\times$ 3, and the number of input channels is eight. Nine lasers with different wavelengths are used to feed the system. Input images are encoded in transmitted intensities of all-pass IM-MRMs by modulation banks. Kernel values are loaded to add-drop IM-MRMs in weight banks, which then perform the dot products with input signals. Finally, all signals are accumulated at the output port, resulting in the convolved feature. Activation functions, pooling, and fully connected layers are followed offline. (**b**) Transmission spectra of nine cascaded add-drop IM-MRMs at through (blue) and drop (red) ports with the correlated mapping value of 1 (dashed curves) and 0.5 (solid curves) in each wavelength channel. The wavelength channel spacing is limited with $\delta \omega$ = 0.5. (**c**) Attainable mapping values for nine cascaded IM-MRMs with the limited wavelength channel spacing. A common range of [–0.5, 0.5] is used for the proposed CNN system. (**d**) Performance of the proposed CNN system on MNIST recognition task versus input precision.

To develop the IM-MRM-based CNN, as shown in Fig. 10a, the input laser beams are first multiplexed using WDM. Then the multiplexed signal is split into separate input channels in parallel with each channel realizing one of the filters. In our developed network 9 all-pass IM-MRMs are cascaded on each input channel, which can be intensity modulated individually to represent a subset of 9 pixels from the input image (28 $\times$ 28 pixels). Considering the 8 different filters of the convolution layer, the convolution part of our architecture comprises 8 input channels, which result to the total of 72 all-pass IM-MRMs that serve as modulation banks for input data encoding. Each input channel is followed by 9 add-drop IM-MRMs, representing the 3 $\times$ 3 kernel to weight the loaded wavelength-multiplex signal from modulation banks, i.e., inputs. Each MRM in weight banks is intensity-modulated at a unique wavelength, in line with operational wavelengths in modulation banks. Finally, output signals of weight banks are accumulated and converted into electrical signals by BPDs at the output of each input channel. Each single convolved pixel is then obtained by adding all electrical signals together through voltage adders. The output of each of the convolutional layer goes through an offline ReLU activation function followed by an average pooling layer with a 2 $\times$ 2 kernel. At the end, two fully-connected layers, with ReLU and Softmax non-linear activation functions, are added to complete the CNN architecture. We would like to note that the weights and biases of the developed CNN are obtained by offline training of the network on a Graphics Processing Unit (GPU) using TensorFlow (Python), while the number of epochs and the batch size are set to 10 and 32, respectively.

Transmission responses of 9 cascaded add-drop IM-MRMs are simulated using the aforementioned custom compact model in Lumerical INTERCONNECT. By evenly adjusting the radius of each MRM, from 15 to 16.6 $\upmu$m, output powers at through (blue) and drop (red) ports are calculated and presented in dashed curves in Fig 10b. The linewidth-normalized channel spacing is set to 0.5 between adjacent wavelength channels to introduce the power penalty crosstalk. In this scenario, the transmission response of each MRM represents the maximum value of 1. From Fig. 6d, we notice that when the mapping value in Channel-1 is set to 1, there is no room for modulating the mapping value in Channel-2. Therefore, to allow a [-0.5, 0.5] mapping range for each wavelength channel, all MRMs need to be pre-adjusted. Solid curves in Fig. 10b show the transmission spectra after pre-adjustment. Nine resonant peaks appear with the mapping value set to 0.5, which is realized by adding the voltage pair to each model. In Fig. 10c, attainable mapping ranges for nine cascaded IM-MRMs are presented as a function of applied voltage pairs by simulation. Compared with the measured voltage pair, simulation models do not involve the thermal crosstalk; thus resulting in different voltage pair results. A common mapping range of [-0.5, 0.5] is selected (red area) for all wavelength channels to realize the 3-dB power penalty in the system, although a larger range of [-0.75, 0.75] is available. Since for Type-II systems (orange curve in Fig. 6e), the maximum attainable value is 0.75 for $\delta \omega$ = 0.5. While the maximum possible range of only 0.5 is achieved for Type-I systems (blue curve in Fig. 6e), which eventually limits the system’s common range to [-0.5, 0.5] when connecting modulation banks with weight banks.

Frequency-domain simulation in Lumerical INTERCONNECT uses scattering data analysis to calculate the overall circuit response. It is done by solving a sparse matrix that represents the circuit as connected scattering matrices, each representing the frequency response of a single element. ^50^. Having 72 IM-MRM elements in both modulation and weight banks separately would result in massive computation to obtain a single transmission response. Furthermore, considering 28 $\times$ 28 pixels in each MNIST input image, convolution calculations using the proposed simulation system in Lumerical INTERCONNECT will be time-consuming by continuous loading of 3 $\times$ 3 input subsets in strides of 1, let alone more than 10,000 MNIST handwritten images are employed for inference. A co-simulation pipeline using Lumerical Application Programming Interface (API) in a Python environment was developed to speed up the CNN simulation. The API can be used for developing scripts or programs via Python that treat Lumerical solvers as clients, and enable users to perform custom analyses, undertake enhanced optimization and visualization, produce plots, and automate complex workflows. Detailed information on the co-simulation pipeline can be found in Section S5 of the Supplementary Information.

By loading the kernel to the Python-Lumerical simulation pipeline, the CNN simulator performs the MNIST recognition task. For every input subset, normalized data is implemented to modulation banks by adjusting the transmission intensity of each all-pass IM-MRM. Then, the signal-encoded light is intensity-modulated by weight banks. Since the same kernel is applied across the set of inputs, weight banks are modified only when a new kernel is loaded. A single convolved pixel output is obtained at the readout in the electrical domain. A voltage is then added to represent the bias. Prediction accuracies versus different encoding precision of the MNIST recognition task solved by the proposed CNN simulator are shown in Fig. 10d. It reveals that with a limited wavelength channel spacing ($\delta \omega$ = 0.5) and mapping range of [-0.5, 0.5], performance for the proposed CNN architecture using IM-MRMs as weight banks can admit > 96.76$\%$ prediction accuracy when applying a 6-bit encoding precision or more. Individual results of the MNIST recognition task with each precision can be found in Figure S10 of the Supplementary Information.

## Discussion and conclusion

Compared to digital accelerators in the latest CMOS processes, optical or optoelectronic MAC operations in SOI-based PIC platforms can process signals with lower latency and higher throughput ^51^. The optical link budget and total energy efficiency are discussed in this section to shed light on the advantages and challenges of implementing MAC operations using our proposed photonic processing unit with IM-MRMs.

**Figure 11:**
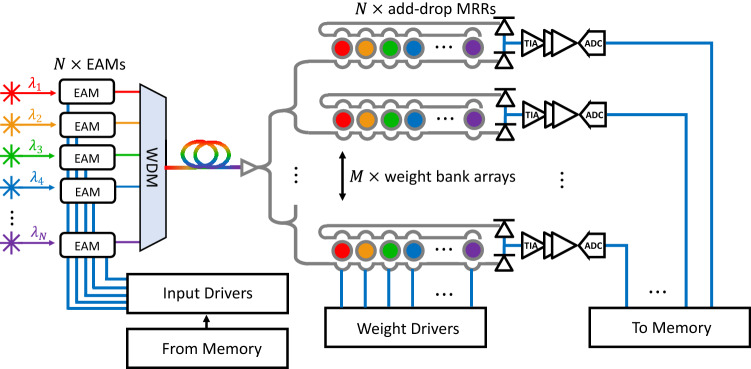
Schematic of the proposed IM-MRM-based $N \times M$ photonic processing system, where a set of high-speed EAMs (or EOMs) are used for input data encoding.

**Figure 12:**
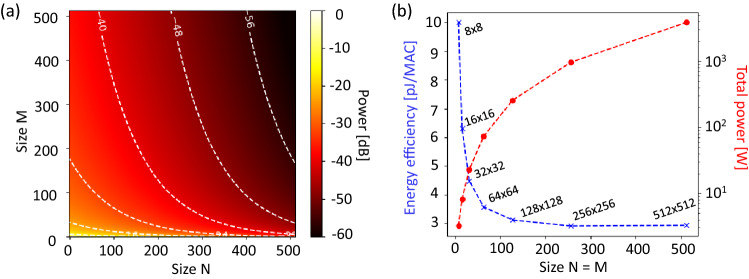
(**a**) Optical link budget of an $N \times M$ photonic processing system with 3-dB inter-channel crosstalk penalty when *N* and *M* vary from 0 to 512. (**b**) Total energy efficiency (blue) and power consumption (red) of a square-size (*N* = *M*) photonic processing system with a fixed precision of 8.5 bits.

In order to analyze the optical link budget and energy efficiency, an $N \times M$ vector-matrix multiplication system is implemented using the proposed IM-MRMs. A frequency comb source is utilized to provide multiple wavelengths from $\lambda _1$ to $\lambda _\text {N}$. The multiwavelength light that includes *N* carrier wavelengths is modulated by an array of high-speed EAMs or EOMs for the input vector encoding and then coupled to the photonic chip. The modulated input is split into *M* branches and weighted by weight banks. Each weight bank array contains *N* add-drop MRR filters. After weight banks, *M* receivers collect the multiwavelength signals and convert them to electrical information for memory access. All-pass IM-MRMs in the modulation bank are replaced with EAMs or EOMs, since the thermo-optic tuning bandwidth of the photoconductive heater (PH) is around 175 kHz ^52^. As a results, (i) the channel density can be further improved ($\delta \omega$ = 0.2 in Fig. 6e), and thus more wavelength channels can be realized ($\sim$580 wavelengths channels for 3-dB power penalty); (ii) the energy consumption can be reduced and (iii) the modulation speed can be enhanced using high-speed EAMs or EOMs. Recently, a high-speed evanescent-coupled Ge waveguide EAM has been proposed with simple fabrication processes on the SOI platform and modulation speed of 56 GHz and dynamic power consumption of 45 fJ/bit ^53^. The schematic of the $N \times M$ photonic processing system is illustrated in Fig. 11. The optical link budget is calculated based on the following equation ^51^:

4 $$\begin{aligned} P_\text {out} = P_\text {laser} - P_\text {EAM} - P_\text {coupling} - P_\text {Si-prop} - P_\text {splitter} - P_\text {IL-MRM} - (N-1)P_\text {OBL-MRM} - P_\text {crosstalk} - P_\text {penalty}, \end{aligned}$$

where $P_\text {laser}$ is the optical power of the laser, $P_\text {EAM}$ is the insertion loss of the EAM, $P_\text {coupling}$ is the loss introduced by the attenuation of the single-mode optical fiber (SMF) and the fiber-to-chip coupling loss, $P_\text {Si-prop}$ is the silicon waveguide attenuation, $P_\text {splitter}$ is the splitter insertion and excess loss, $P_\text {IL-MRM}$ is the insertion loss of the add-drop IM-MRR filter at the input vector wavelength, $P_\text {crosstalk}$ is the inter-channel crosstalk penalty, and $P_\text {OBL-MRM}$ is the out-of-band insertion loss when the resonant peak is not aligned with input wavelengths, and $P_\text {penalty}$ is the network penalty due to extinction ratio, crosstalk, and laser relative intensity noise (RIN). Considering $P_\text {laser}$ = 0 dBm, $P_\text {coupling}$ = 1.6 dB ^51^, $P_\text {EAM}$ = 6.2 dB ^53^, $P_\text {Si-prop}$ = 2.5 dB/cm $\times$ $3(N-1)L$ where *L* in centimeters is the waveguide spacing between the two MRMs, $P_\text {splitter}$ = 3.3 $\times$ log$_2M$ dB, $P_\text {IL-MRM}$ = 1.25 dB, $P_\text {crosstalk}$ = 3 dB, $P_\text {OBL-MRM}$ = 0.01 dB ^51^, and $P_\text {penalty}$ = 4.8 dB ^51^, the optical link budget as a function of the dimensions of the proposed photonic processing system is calculated and plotted in Fig. 12a. The total length of the waveguide is approximated as the length spanning the optical depth of the system, where the distance between two adjacent MRMs is set to 50 $\upmu$m to minimize the thermal crosstalk. As can be seen in Fig. 12a, the number of MRMs (*N*) in each array does not have a huge impact on the link budget, since only one MRM aligned with the input wavelength contributes 1.25-dB to the insertion loss, but the remaining out-of-band MRMs each contribute 0.01 dB. For *M* weight bank arrays, the attenuation introduced due to log$_2M$ splitters degrades the optical power and poses a limitation on the energy efficiency. In the optical link budget calculation, we estimate around 60 dB of loss for a 512 $\times$ 512 tensor core based on the proposed IM-MRR system. The loss can be compensated by either increasing the laser’s output power or adding SOAs to the system. It is worth mentioning that whole SOAs can pre-amplify the input signal, the non-linear gain-current curve would need to be calibration for ^51^.

The total energy consumption of the proposed system to overcome the shot noise and the capacitance of the PD at the output with a fixed n-bit precision can be expressed by the following equation:

5 $$\begin{aligned}{} & {} P_\text {total} = N (\frac{P_\text {laser}}{\rho _\text {SOA}}) + \frac{N}{M}\frac{hv}{\eta }\text {max}(2^{2n+1}, \frac{C_\text {d}V_\text {r}}{e})f_\text {mod} + N(E_\text {EAM}\times n)f_\text {mod} \nonumber \\{} & {} \quad \quad \quad \,\,+ NM\times P_\text {MRM} + N \times k P_\text {SOA} + M \times P_\text {rec}, \end{aligned}$$

where, *hv* is the photon energy for a center wavelength of 1550 nm, $C_\text {d}$ = 2.4 fF and $V_\text {r}$ = 1 V are the capacitance and driving voltage of the PD, respectively, and $\eta$ is the quantum efficiency of the detector ($\eta _\text {PD}$ = 80%), laser ($\eta _\text {laser}$ = 20%) and the optical loss through the $N \times M$ system. Considering the noise equivalent power of 0.214 pW/$\sqrt{HZ}$ at the readout, the total required power for 8.5-bit precision needs to be more than -22.6 dB with the sample rate of 5 GS/s. Based on the calculations done in Fig. 12a, this limits the size of the system to 20 $\times$ 20. Hence, SOAs are required in the system to compensate for the loss. $\rho _\text {SOA}$ is the efficiency enhancement due to an SOA ($\rho _\text {SOA} = 10^{G/10}$, where *G* is the gain of the SOA ^51^). The modulation speed of the system is denoted by $f_\text {mod}$. The energy associated with signal modulation and detection is dominated by EAMs ($E_\text {EAM}$ = 45 fJ/bit), add-drop IM-MRMs ($P_\text {MRM}$ = $I_\text {IRPH}V_\text {IRPH}$+$I_\text {ICPH}V_\text {ICPH}$) and ADCs ($P_\text {rec}\approx$ 200 mW). Assuming the system’s sample rate is 5 GS/s limited by ADCs and DACs with the input laser power of 10 dBm and an SOA gain of 17 dB ^54^, the total energy efficiency (Joule per MAC) and total power consumption of an $N \times M$ photonic processing system with a fixed 8.5-bit precision are calculated and plotted in Fig. 12b. To enable 512 $\times$ 512 implementation, two SOAs are required in each channel ($k = 2$ in Equation 5). To clarify, the calculation does not include the power consumed by the temperature control system, which consumes 36 W in operation. The energy efficiency (dashed blue curve) increases with matrix size due to the fact that more MAC operations can be handled simultaneously in larger systems. It has been observed that photonic tensor cores outperform electronic counterparts as the matrix size exceeds 500 ^55^. However, the total power consumption (dashed red curve) surges simultaneously since add-drop IM-MRR filters for weight banks consume considerable power ($P_\text {MRM}$ = $\sim$13.3 mW). One of the solutions is replacing the PH with low power index-modulation components, such as non-volatile PCMs in the weight bank. For PCMs, such as GST ^56^ and GSST ^57^, the refractive index (*n*) and the extinction coefficient (*k*) increases when the material changes its phase from amorphous to crystalline. Hence, they generate high absorption losses. Recently, a wide bandgap PCM Sb$_2$S$_3$ that allows for a strong optical phase modulation and low optical loss was experimentally demonstrated on the SOI platform at 1550 nm, with a figure of merit (FOM = $\Delta n/\Delta k$) of 10.8 ^58^, which can be a potential candidate for our future design.

In summary, we have demonstrated a novel photonic processing unit using the intensity-modulation-based MRM. By utilizing an unbalanced MZI serving as a tunable coupler to couple the light into the ring resonator, the ER of the resonant peak can be directly modulated by changing its coupling strength. To lock the resonant peak at a fixed wavelength during modulation, another index modulator is implemented in the resonator to offset the wavelength shift during the ER modulation. Using the intensity modulation scheme at a fixed wavelength, we demonstrated that the wavelength channel spacing with 3-dB power penalty crosstalk tolerance is 17-fold denser than the conventional wavelength-modulated counterpart. As a result of the increased channel density, our proposed system allows up to 578 wavelength channels with a 3-dB power penalty when weight banks are designed using IM-MRMs with a radius of 5 $\upmu$m. A photonic dot product core was presented for the proof-of-concept demonstration. The photonic chip, containing one IM-MRM serving as the input encoder and one IM-MRM serving as the weight encoder, was integrated on a PCB through an electrical wire bonding/photonic wire bonding (EWB/PWB) co-packaging technique. Applying the “discrete analog” encoding/decoding scheme, a 3-bit input and 4-bit signed weighting dot product computation was realized in the optical domain. One thousand random decimal floating-point dot product results showed an MSE 3.09 $\times$ 10$^{-3}$, experimentally demonstrating the capability of our proposed IM-MZMs for optical information processing.

Future work will focus on: (i) optimizing the IM-MRM design to reduce the thermal crosstalk between the ICPH and the IRPH, such as moving the IRPH to the opposite side of the MRR and using a separate ground; (ii) replacing photoconductive heaters with non-volatile PCMs in the IM-MRR filter for weighting; (iii) decreasing the energy consumption and improving the modulation bandwidth of the system by replacing modulation banks with directly modulated lasers or high-speed EAMs and EOMs.

## Supplementary Information

Supplementary Information.

## Data Availability

Data underlying the results presented in this paper are not publicly available at this time but may be obtained from the authors upon reasonable request.
