# Supplementary material for: Towards a high-density photonic tensor core enabled by intensity-modulated microrings and photonic wire bonding
Source: Sci Rep. 2023 Jan 23;13:1260. doi: 10.1038/s41598-023-27724-y (PMC9870901; doi:10.1038/s41598-023-27724-y)
Supplement: Supplementary file 1 — Supplementary Information. [file 41598_2023_27724_MOESM1_ESM.docx]

**Supplementary Information for:**

Towards a High-density Photonic Tensor Core Enabled by Intensity-modulated Microrings and Photonic Wire Bonding

Enxiao Luan^1,2*^, Shangxuan Yu^2^, Mahsa Salmani^1^, Mohammadreza Sanadgol Nezami^2^, Bhavin J. Shastri^3^,
Lukas Chrostowski^2^, and Armaghan Eshaghi^1^

^1^Huawei Technologies Canada Co., Ltd. 19 Allstate Parkway, Markham, Ontario, L3R 5A4, Canada

^2^Department of Electrical and Computer Engineering, The University of British Columbia, 2332 Main Mall,
Vancouver, BC, V6T 1Z4, Canada

^3^Department of Physics, Engineering Physics & Astronomy, Queen’s University, Kingston, ON KL7 3N6, Canada

[*eluan@ece.ubc.ca](mailto:*eluan@ece.ubc.ca)

1. **Design of the Proposed Modulator**

The design of the proposed intensity-modulation-based microring modulator (IM-MRM) follows standard design rules of the microring resonator (MRR) described in [1], except that a two-point Mach-Zehnder interferometer-based (MZI) coupler is utilized for the ring-bus coupling. Transfer Matrix Method (TMM) is used to calculate the transmission and reflection between the MZI and MRR, as shown in Figure S1(a). Equations (1) and (2) shown below present the transfer matrix and transfer function between the input ($E_{in1}$ and$E_{in2}$) and output ($E_{thru1}$ and$E_{thru2}$) electric fields of the MZI [2]:

|  | $\left[ \begin{matrix} E_{thru1} \\ E_{thru2} \end{matrix} \right]=\left[ \begin{matrix} T_{1} & K_{2} \\ K_{1} & T_{2} \end{matrix} \right]\left[ \begin{matrix} E_{in1} \\ E_{in2} \end{matrix} \right]$, | (1) |
| --- | --- | --- |
|  | $E_{in2}=\alpha_{MRR}exp(-j\delta)E_{thru2}$, | (2) |

where $\alpha_{MRR}$ represents the single-pass amplitude transmission and $\delta=\beta L$ represents the single-pass phase shift in the MRR, $\beta$ is the propagation constant and $L=2\pi R,$ and $R$ is the radius of the MRR. $T_{i}$ and $K_{i}$ represent the transmission from the $i^{th}$ input port of the MZI to the through-port and cross-port output ($i$ = 1, 2), respectively, and can be calculated using the following equations:

|  | $T_{1}=t_{1}t_{2}\alpha_{arm1}\exp\left( -j\left( \phi_{1}+\Delta\phi_{1} \right) \right)+k_{1}k_{2}\alpha_{arm2}\exp\left( -j\phi_{2} \right)$, | (3) |
| --- | --- | --- |
|  | $T_{2}=t_{1}t_{2}\alpha_{arm2}\exp\left( -j\phi_{2} \right)+k_{1}k_{2}\alpha_{arm1}exp(-j(\phi_{1}+\Delta\phi_{1}))$, | (4) |
|  | $K_{1}=k_{1}t_{2}\alpha_{arm2}\exp\left( -j\phi_{2}) \right)+t_{1}k_{2}\alpha_{arm1}\exp\left( -j\left( \phi_{1}+\Delta\phi_{1} \right) \right)$, | (5) |
|  | $K_{2}=k_{1}t_{2}\alpha_{arm1}\exp\left( -j\left( \phi_{1}+\Delta\phi_{1} \right) \right)+t_{1}k_{2}\alpha_{arm2}exp(-j\phi_{2})$. | (6) |

where $t_{i}$ and $k_{i}$ represent the transfer functions of the electric field coupled to the through-port and cross-port of the $i^{th}$coupler ($i$ = 1, 2), respectively, and $\phi_{1}$ and $\phi_{2}$are the phase responses of the upper and lower MZI arms. $\Delta\phi_{1}$ represents the phase change induced by manipulating the index modulation element in the upper arm of the MZI coupler, and $\alpha_{arm1}$ and $\alpha_{arm2}$ are the amplitude transmissions, accordingly. The electric field transfer function at the through port of the device is [2]:

|  | $\frac{E_{thru1}}{E_{in1}}=\frac{T_{1}-(T_{1}T_{2}-K_{1}K_{2})\alpha_{MRR}exp(-j(\delta+\Delta\phi_{2}))}{1-T_{2}\alpha_{MRR}exp(-j(\delta+\Delta\phi_{2}))}.$ | (7) |
| --- | --- | --- |

If we assume a loss-less MZI coupler in the design:

|  | $T_{1}T_{2}-K_{1}K_{2}=exp(-j(\phi_{1}+\phi_{2}+\Delta\phi_{1}))$, | (8) |
| --- | --- | --- |
|  | $T_{1}=sin((\phi_{1}-\phi_{2}+\Delta\phi_{1})/2)exp(-j(\phi_{1}+\phi_{2}+\Delta\phi_{1}+\pi)/2)$, | (9) |
|  | $T_{2}=sin((\phi_{1}-\phi_{2}+\Delta\phi_{1})/2)exp(-j(\phi_{1}+\phi_{2}+\Delta\phi_{1}-\pi)/2)$. | (10) |

Therefore, according to Equations (7) and (8), the resonance condition (when $\phi_{1}+\phi_{2}+\Delta\phi_{1}+\delta+\Delta\phi_{2}=2\pi m$, where $m$ is an integer) of the device depends on the variation of both phase changes $\Delta\phi_{1}$ and $\Delta\phi_{2}$. While, the transmission amplitude, according to Equations (9) and (10), depends on the additional phase change ($\Delta\phi_{1}$) between MZI’s two arms. The interference modifies the effective coupling ratio in the MZI coupler, thus changing the extinction ratio (ER) at the resonant peak.

As shown in Figure S1(a), in our proposed design, an unbalanced MZI coupler is used for input/output coupling. Therefore, the coupling coefficient is wavelength-dependent. When a broadband phase shift $\Delta\phi_{1}$ on the upper arm of the MZI is actuated, the sinusoidal spectral response of the MZI coupler shifts in wavelength, thereby changing the effective coupling ratio to the MRR [3]. At the same time, the resonance condition will change accordingly. Therefore, the index modulation element in the MRR serves as the wavelength drift compensator to offset the drift of the resonant peak with a negligible impact on the transmission amplitude.


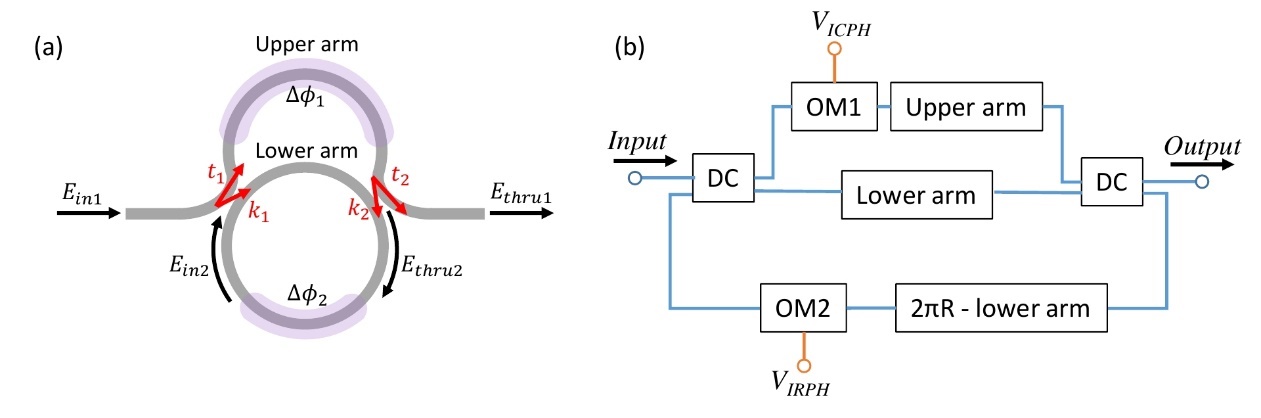


Figure S1. (a) Schematic of the proposed IM-MRM. Purple areas represent index modulation elements, providing $\Delta\phi_{1}$ and $\Delta\phi_{2}$ phase shifts to the MZI coupler and the MRR, respectively. (b) Block diagrams of the proposed IM-MRM custom compact model in INTERCONNECT. Two DC blocks are used to couple the light on and off the MRR, two OM elements are used for the index modulation, and 3 passive waveguide elements are used to build the MZI and MRR. Blue lines represent optical interconnects and orange lines represent electrical interconnects.

In our proposed design (as shown in the block diagrams in Figure S1(b)), the index modulation element in the MRR has been placed in the lower arm of the MZI to balance the refractive indices between two arms and to reduce the footprint by sharing the ground. This design realizes the intensity modulation at a fixed wavelength, but thermal crosstalk between the upper and lower arms is observed. Moreover, modulating the index modulation element in the lower arm not only changes the resonance condition but changes the effective coupling ratio in the MZI coupler, thus changing the ER slightly. Luckily, it happens with lower efficiency compared with the upper arm’s modulation efficiency which has a longer length. In the future design, this will be optimized.

The simulation model for the proposed IM-MRM was developed using Lumerical Suite [4]. Lumerical MODE Solutions and DEVICE were first used to obtain the effective index change and loss as a function of bias voltage for the N-doped resistive heater. The data was then imported to INTERCONNECT to build the optical modulator (OM) element with parameterizable lengths and bias voltages. Figure S1(b) shows block diagrams of the custom compact model for the proposed all-pass IM-MRM. Two directional coupler (DC) blocks and three passive waveguide sections, as well as two OM elements, were used to build the MZI coupler and the MRR. It is worth noting that the DC and the passive waveguide are parameterizable. Therefore, the compact model can be adjusted with the coupling strength, the lengths of the MZI coupler arms, the radius of the MRR, and the fill-factor of the N-doped resistive heater to obtain the best performance. At last, we changed the applied voltages, $V_{ICPH}$ and $V_{IRPH}$, independently and recorded the resonance wavelength drift accordingly to find the wavelength compensation voltage pair for locking the resonant peak of the model. By applying the obtained voltage pairs to $V_{ICPH}$ and $V_{IRPH}$ ports, the intensity modulation at a fixed wavelength is realized.

1. **Device Characterization Setup and Linear Predistortion**

The proposed IM-MRM device intensity modulation measurement and characterization were performed on a custom silicon photonic test setup as shown in Figure S2(a) and S2(b). The optical measurement setup consists of a tunable off-chip laser source with power meters as the detector, an optical fiber array to couple the light on and off the chip, and a thermally-tuned stage controlled by a temperature controller (TEC). Electrical control of ICPH and IRPH were performed by a source meter in the constant voltage mode.


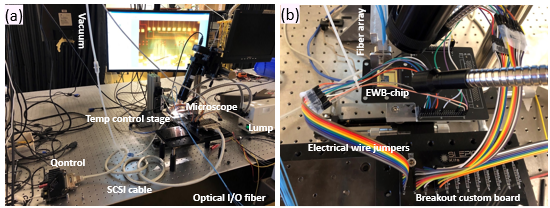


Figure S2. (a) The measurement setup for the co-packaged dot product photonic chip on the PCB. (b) Zoomed-in plot of the chip with electrical and optical I/Os.

Since our proposed IM-MRM is operated leveraging the thermo-optic effect, the relationship between the applied voltage and the transmitted power is non-linear. The non-linear response of the detected transmitted powers is obtained and shown in Figure S3(a) by sweeping the voltage pairs with a fixed $V_{ICPH}$step. The power levels are collected at the minimum and maximum of the power range with 4-bit precision. To obtain a linear relationship between the applied voltage pair and the transmitted power, a predistortion step is introduced. As depicted in Figure S3(b), according to the obtained detected power, a polynomial interpolation method is introduced which adds more points along the non-linear curve. By re-distributing 16 points with a fixed transmitted power level (yellow dots in Figure S3(b)), the required $V_{ICPH}$values are obtained. By applying the re-distributed voltage pair, generated by the predistortion step, to the IM-MRM, sixteen linearly distributed power levels are observed in Figure 4(c) of the main paper. This is important for the implementation of linear encoding/decoding with multiple wavelength channels.

**
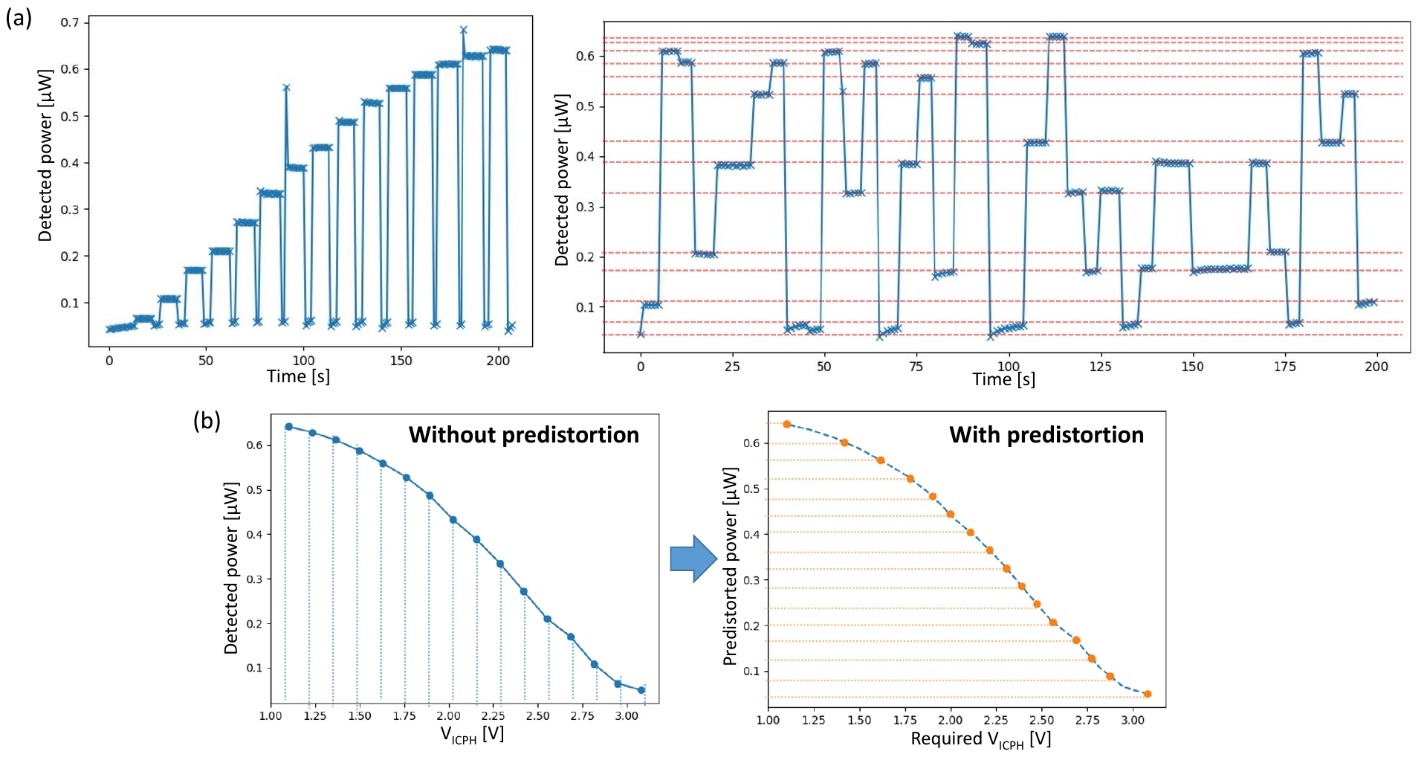
**

Figure S3. (a) 16 power levels in consecutive ascending order of the all-pass IM-MRM. A non-linear relationship between the detected power level and the applied voltage pair is observed. (b) 16 power levels vs. applied voltages without and with the predistortion step. After predistortion, linearly distributed 16 power levels are observed.

1. **Coherent Interference within Multiple Cascaded MRRs**

The analysis of multiple cascaded MRRs behavior for investigating the coherent interference follows the method in Ref. [5]. Figure S4(a) illustrates the block diagram of the single add-drop MRR transfer function generated by using the 4-port transmission line couplers introduced in [6]. Two couplers used to model the coupling between the bus waveguide and MRR contain two parameters $(\gamma_{c}, K_{i})$. $\gamma_{c}$ is the insertion loss and $K_{i}$is the coupling coefficient. $L_{1}$ and $L_{2}$ represent the transmission line pair of the MRR with the attenuation factor of $\alpha_{p}$. Figure S4(b) shows the combination of the single add-drop MRR model with ports 1 and 3 as the input and through ports and ports 2 and 4 as the drop and add ports, which can be utilized in the cascaded MRR system.


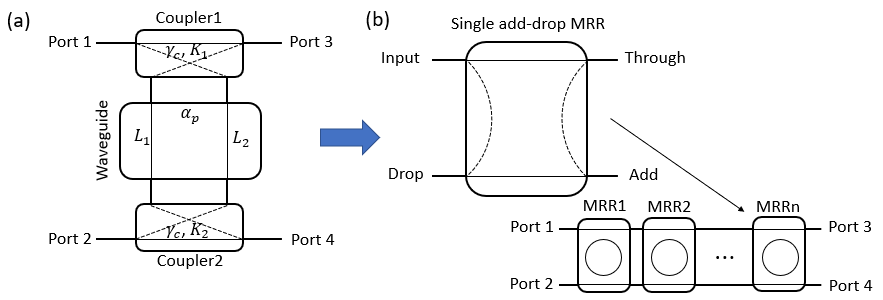


Figure S4. (a) Block diagram of the 4-port transmission line coupler for a single add-drop MRR model following the method in Ref. [5, 6]. (b) Block diagram of a combination of coupler units to model an MRR filter, which can be used in the cascaded MRR system.

To simplify the analysis, we assume two couplers are identical in the add-drop MRR ($K_{1}=K_{2}=K$). Therefore, the transmission at the through ($T_{1MRR}$) and drop ($D_{1MRR}$) ports of the single add-drop MRR can be expressed as [5]:

|  | $T_{1MRR}=\gamma_{c}\sqrt{1-K}\cdot\left( 1-FK \right)$, | (11) |
| --- | --- | --- |
|  | $D_{1MRR}=-\gamma_{c}^{2}K\cdot e^{-\left( \alpha_{p}+j\beta\right)L}\cdot F$, | (12) |

where $F={[1-G\cdot exp(-j\beta L)]}^{-1}$ represents the cavity-resonance amplification factor, $G= \gamma_{c}^{2}\left( 1-K \right)exp(-\alpha_{p}L)$ represents the round-trip amplitude loss, $\beta$ is the propagation constant, and $L=L_{1}+L_{2}$ is the cavity length of the MRR. When $F$ is maximum, the interference factors for through ($1-FK$) and drop ($F$) ports reach their minimum and maximum. Thus, the transmission amplitude is minimized and maximized at the MRR’s through and drop ports, respectively (on resonance).

For the two cascaded add-drop MRRs system, the bus waveguides connecting the two MRRs (MRR-A and MRR-B) on the through and drop sides are with an equal length of $\Delta L$ for simplification. The through port and drop port transmission can be expressed as [5]:

|  | $T_{2MRR}=T_{A}T_{B}\cdot e^{-(\alpha_{p}+j\beta)\Delta L}F_{AB}$, | (13) |
| --- | --- | --- |
|  | $D_{2MRR}=D_{A}+T_{A}^{2}D_{B}\cdot e^{-(\alpha_{p}+j\beta)\cdot2\Delta L}F_{AB}$, | (14) |

where $F_{AB}={[1-D_{A}D_{B}\cdot exp\left( -(\alpha_{p}+j\beta)\cdot2\Delta L \right)]}^{-1}$ is introduced. In Equation 13, we observe that the through port transmission of the two cascaded MRRs system ($T_{2MRR}$) reaches the minimum at each resonance wavelength for MRR-A and MRR-B, respectively. For the drop port, the electric field amplitude is the coherent sum of the two dropped signals from MRR-A and MRR-B, which reaches the maximum on each resonance wavelength in drop port transmission. A validation model is developed with two cascaded add-drop IM-MRR compact models, where the two bus waveguides are phase modulated identically. The simulated drop port transmission in Figure S5 indicates that the amplitude on individual resonant peaks is not impacted by the phase change in bus waveguides, which agrees with the measurements in Ref. [5].


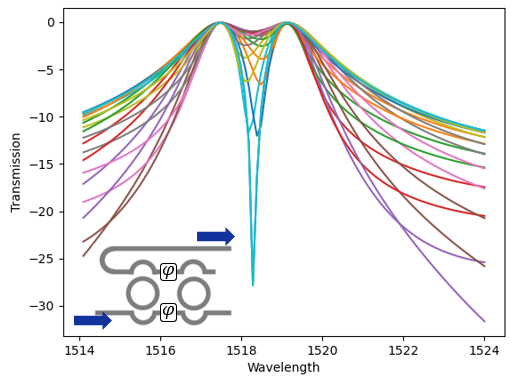


Figure S5. Simulated drop port transmission spectra of two cascaded add-drop IM-MRRs using Lumerical INTERCONNECT. Two phase change elements are employed in bus waveguides to change the phase response from 0 to 2$\pi$ symmetrically.

Furthermore, for the N cascaded add-drop IM-MRRs system, coherent interference due to the resonance of MRRs takes effect with extra resonance factors between MRR-A and MRR-C, or MRR-A and MRR-D etc. [5]. Among all these factors, the one contributing the strongest cross-talk is the MRR presenting the closest resonant peak to the target resonance wavelength (e.g., MRR-A’s resonant peak), since the attainable value for MRR-A is determined by $\max\left[ D_{AB},D_{AC}, D_{AD},D_{AE}\cdots\right]$, where $D_{Ai}$ represents the drop port transmission of MRR-i at MRR-A’s resonance wavelength.

1. **Attainable Values vs. Wavelength Channel Spacing**

As described in Section 2 of the main paper, a custom compact model is developed for the proposed IM-MRM design using Lumerical INTERCONNECT to investigate the inter-channel crosstalk penalty. To visualize the power penalty vs. the wavelength channel spacing, we built two cascaded types (Type-I and Type-II) in INTERCONNECT and simulated with different wavelength channel spacing between the two resonant peaks. The wavelength channel spacing was modified by changing the radius of one of the IM-MRM models. Before the power penalty investigation, the intensity modulation capability of the individual IM-MRMs was tested by applying different voltage pairs. The simulated transmission response at the resonant peak was recorded accordingly and then used to build the relationship between the applied voltage pair and the transmitted power of each IM-MRM model.

Figure S6 presents simulated attainable value ranges for Channel-1 and Channel-2 with different wavelength channel spacing. Figure S6(a) and S6(b) represent the simulation results for Type-I and Type-II systems, respectively. For Type-I systems, the 3-dB penalty tolerable wavelength channel spacing is roughly $\delta\omega$ = 0.5 due to its Lorentzian-shaped resonant peak resulting from the all-pass IM-MRM (see Figure 6(b) of the main paper). While for Type-II systems, thanks to the drop port transmission, the filtered power at the through port in Channel-1 can be detected at the drop port (see Figure 6(c) of the main paper), thus offering a smaller wavelength channel spacing ($\delta\omega$ = 0.2) with 3-dB power penalty.


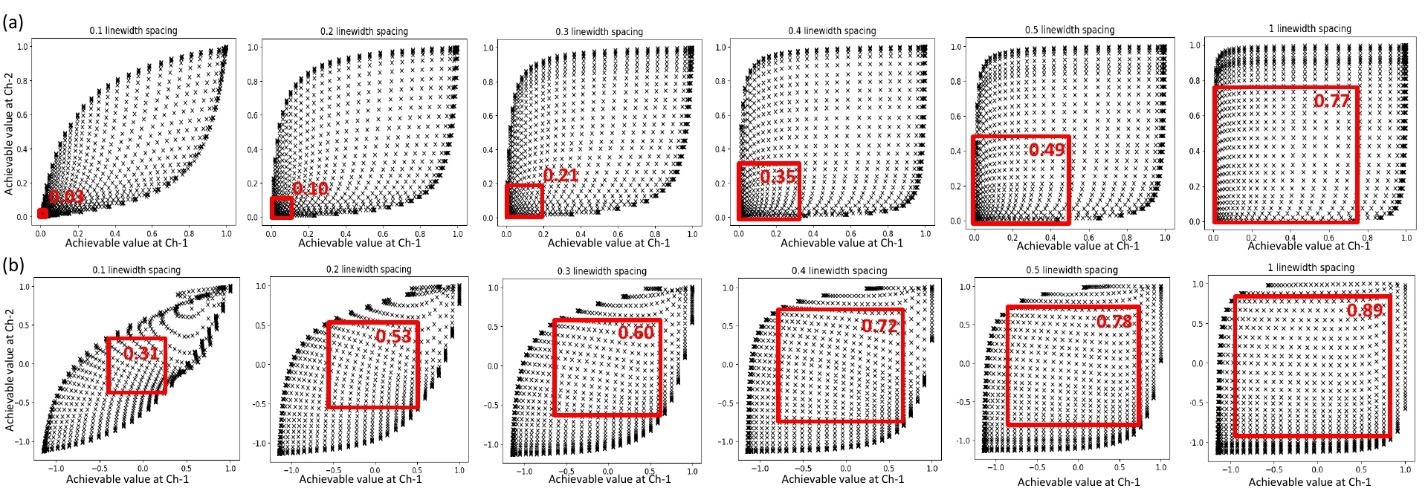


Figure S6. Simulated attainable values for Channel-1 and Channel-2 of two different types of cascaded IM-MRM systems. (a) Type-I system. (b) Type-II system. The red box in the plot represents the usable range.

Similarly, the attainable value range for wavelength-modulation-based MRM systems is investigated and presented in Figure S7. After replacing the IM-MRM model with the standard MRM model in INTERCONNECT, the transmitted power at Channel-1 and Channel-2 were recorded when wavelength channel spacing varies from 0.5$\delta\omega$ to 4$\delta\omega$. Only the Type-II system is considered in the WM-MRM system. As can be seen, due to the wavelength drift, a larger wavelength channel spacing ($4>\delta\omega>3$) is required for the 3-dB power penalty tolerance, which is in good agreement with simulation results in Ref. [7]. For $\delta\omega$ = 0.5, there is no usable range due to the flat-top transmission at the drop port (first plot of Figure S7(a)) which is caused by the coherent interaction between the two adjacent resonant peaks.


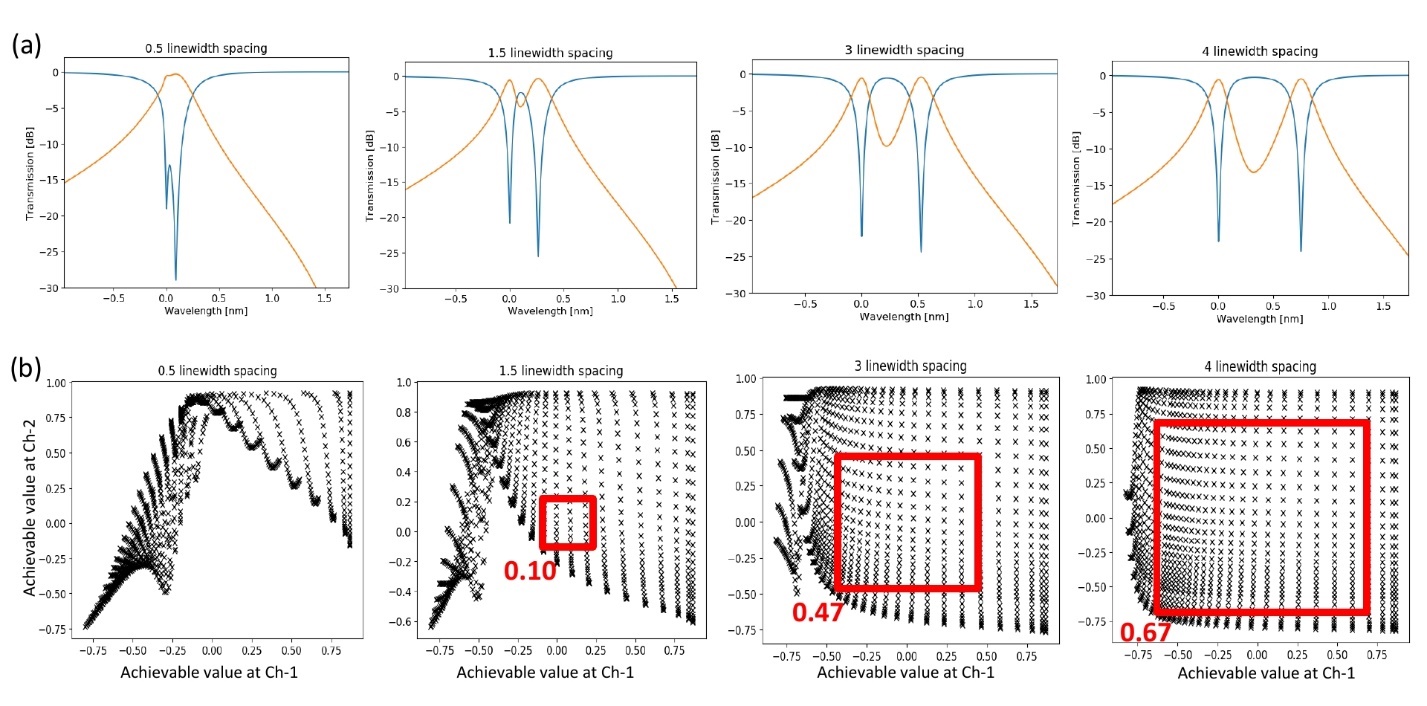


Figure S7. (a) Simulated transmission spectra of Type-II WM-MRM systems with different wavelength channel spacing. Blue curves represent through-port transmissions and orange curves represent drop-port transmissions. (b) Simulated attainable values for Channel-1 and Channel-2 of WM-MRM systems with different wavelength channel spacing. The red box in the plot represents the usable range.

1. **Co-simulation Pipeline and Hand-Written Digit Recognition Results**

To reduce the simulation time for hand-written digit recognition tasks, a co-simulation pipeline using Lumerical API is developed. The schematic of the co-simulation pipeline is presented in Figure S8. We use the aforementioned IM-MRM model in INTERCONNECT to build a convolution channel with WDM incoming signals to add the inter-channel crosstalk penalty between cascaded IM-MRM models. Transmission responses of IM-MRMs in modulation banks and weight banks are extracted for each model as a function of the voltage pairs applied for intensity modulation. Trained weights and biases (calculated using TensorFlow) for the MNIST dataset are rounded to available values according to the transmission response of the weight banks obtained from INTERCONNECT and stored in Python. The rounding accuracy depends on the precision of the weight banks. For inference, new MNIST images are imported through the modulation banks, then weighted by the weight banks for matrix multiplication. The results are obtained by applying the ReLU activation function and performing the remaining operations in average pooling and fully-connected layers. Compared with completing all the simulations in INTERCONNECT, using a co-simulation pipeline can speed up the CNN simulation, especially for large-scale systems. Moreover, this pipeline can incorporate the inter-channel crosstalk penalty into the simulations, thus providing more realistic transmission responses for a cascaded system.


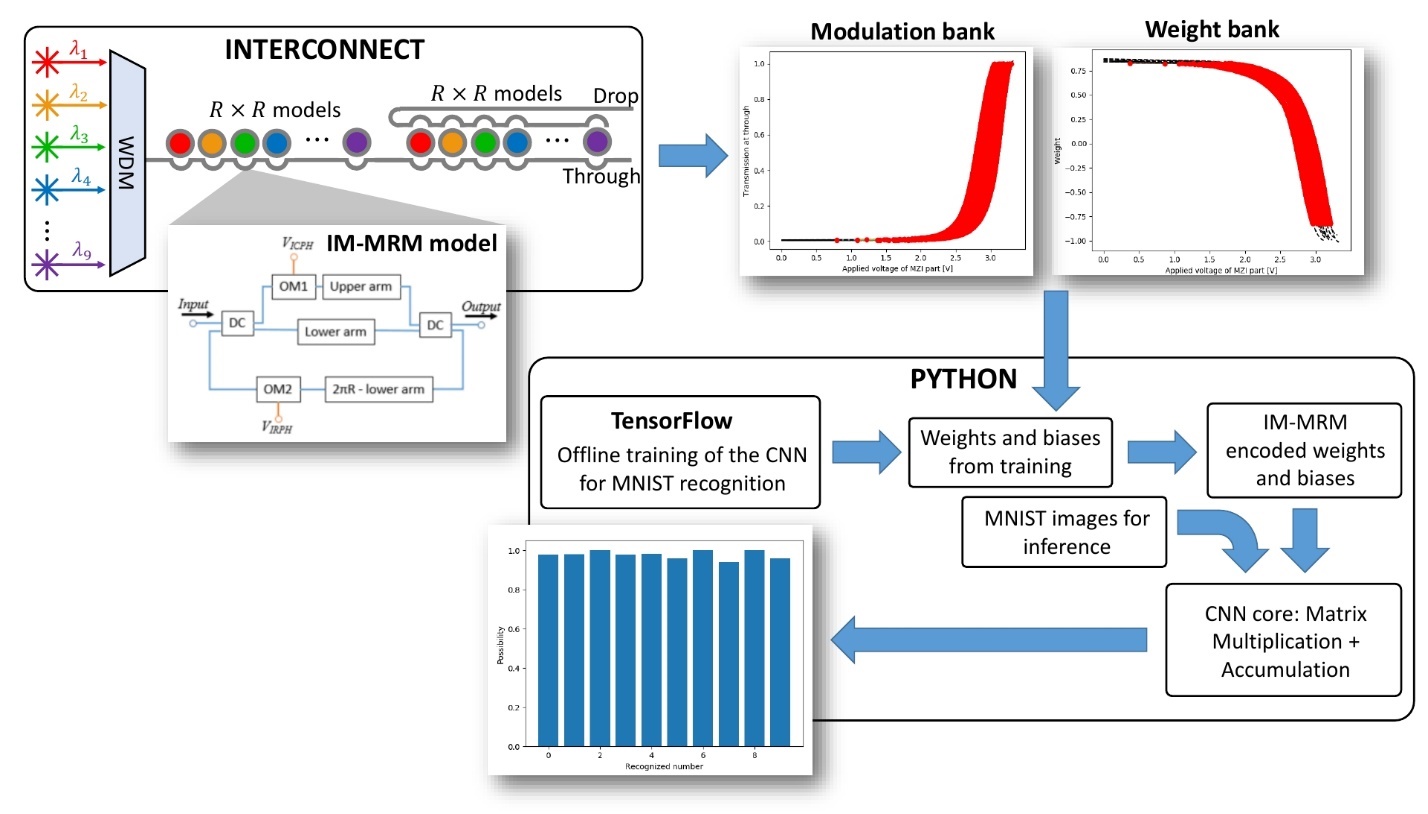


Figure S8. Co-simulation pipeline used for hand-written digit recognition task.

The working procedure of the co-simulation pipeline is as follows. According to the dimensionality ($R\times R$) of the kernel in each channel (the number of kernels is $K$), $R^{2}$Add-drop IM-MRM models are implemented and cascaded with varied radii in Lumerical INTERCONNECT via Python API. The radius of each model is adjusted depending on its operational wavelength from $R^{2}$ light sources. Transmission responses including the inter-channel crosstalk of the cascaded system are calculated in INTERCONNECT and then exported to Python. By applying different voltage pairs to individual IM-MRM models in the cascaded system, the simulated power range is obtained by subtracting transmission responses from drop and through ports for each wavelength (as shown in Figure S9). A common power range is extracted according to the maximum attainable value for all models in the system (see Figure 10(c) of the main paper). The common power range is then normalized and quantized based on the required precision and stored for encoding the kernel in Python. The relationship between each normalized power value and the applied voltage pair for individual IM-MRMs is saved as the mapping table which will be tracked when loading the target value to the system. Similarly, transmission responses of modulation banks are calculated for $R^{2}$all-pass IM-MRM models and used for encoding input WDM signals. Convolution computations for the proposed CNN simulator are proceeded through matrix-matrix multiplication in Python. The subset of the input image is encoded as an input vector using the transmission data of the all-pass IM-MRM, and then multiplied by the kernel vector encoded using the weighting data of the add-drop IM-MRM. The single convolved result is generated by accumulating multiplication outputs and biases from $K$ channels. By striding along the $H\times W$ input image with a stride of $S$, the image is transformed into a matrix of dimensionality of $KDR^{2}\times\left\lceil\frac{H-R}{S}+1 \right\rceil\left\lceil\frac{W-R}{S}+1 \right\rceil K$, where $H$ and $W$ are the height and width of input image including padding, and $D$ is the number of input channels. Because only normalized input values smaller than 1 can be encoded using the modulation banks, the convolution result is first normalized and then goes through a ReLU activation function in Python. After the activation function, the image will pass through the second set of convolutional layer and activation (ReLU) function. A $2\times2$ average-pooling layer is utilized for invariance and direct down-sampling of the convolved features. Finally, a fully-connected layer is fed by flattening the pooled image, and the resultant vector is proceeded with the last fully-connected layer, where the result of the hand-written digit recognition task is obtained using SoftMax function. Convolutional parameters are summarized in Table S1.


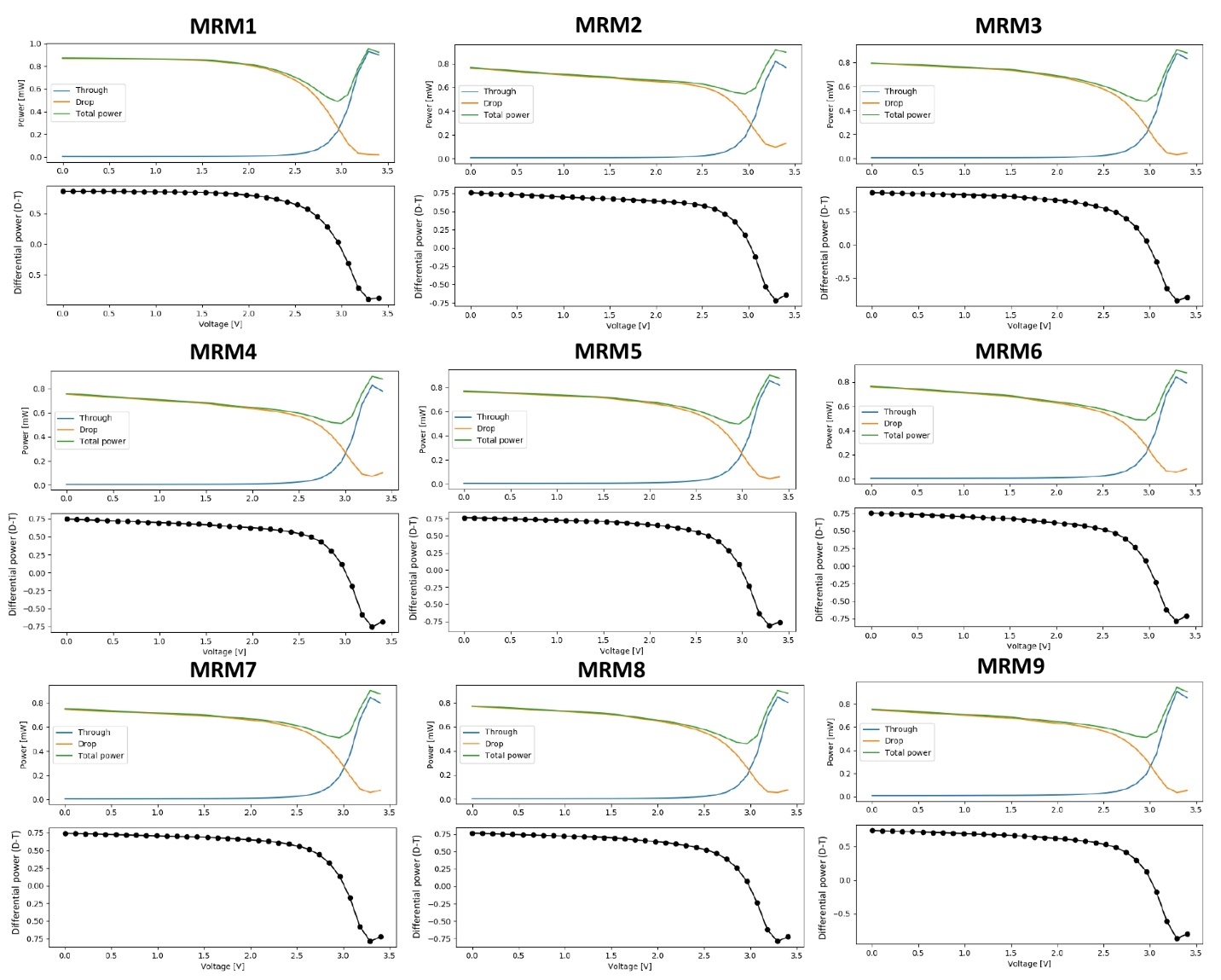


Figure S9. Simulated transmitted power at through and drop ports, and differential power range vs. applied voltage pairs for the intensity modulation of 9 add-drop IM-MRM models in the cascaded system in INTERCONNECT. Blue curves represent the detected power at the through port, and orange curves represent the detected power at the drop port. Green curves show the sum of the detected power at both through and drop ports, indicating the IL of each IM-MRM. Black dots show the differential power calculated by subtracting the drop- and through–port powers for different applied voltage pairs.

Table S1. Summary of convolutional parameters [8]

| **Parameters** | **Meaning** |
| --- | --- |
| *H* | Height of input images including padding |
| *W* | Width of input images including padding |
| *D* | Number of input channels |
| *R* | Kernel’s edge length |
| *K* | Number of kernels |
| *S* | Stride |

Different precision of the transmission responses of the modulation and weight banks in the co-simulation pipeline (with 3-dB power penalty crosstalk), results in different overall accuracy. As shown in Figure S10, when the number of precision bits are changed from 3 to 10, the recognition accuracy increases. Considering the 8.5-bit precision recently achieved using IRPH-based MRMs, our proposed IM-MRM system shows an overall accuracy of ~98%.


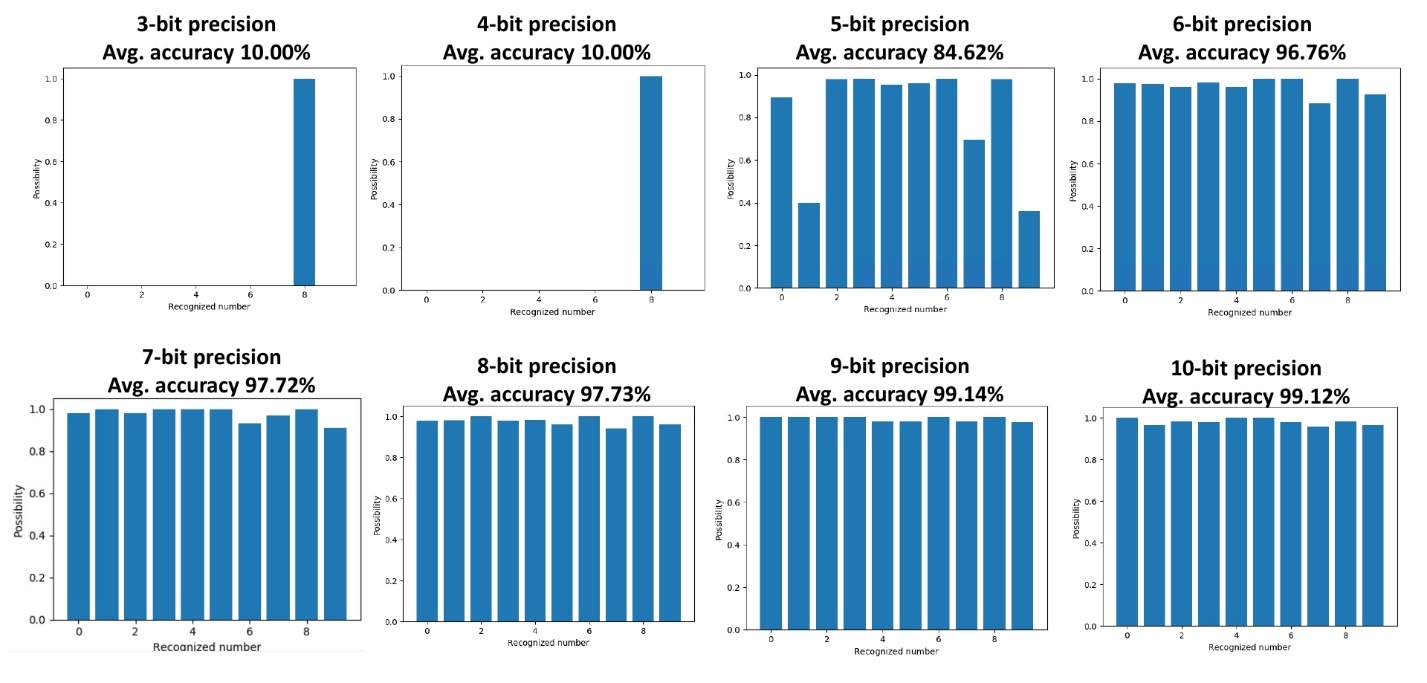


Figure S10. Simulated accuracies of the hand-written digit recognition task for different bits of precision.

1. **Co-packaging of the Photonic Chip and its Measurement Setup**

The UV glue is used to fix the chip and fiber array to the PCB, which is the first step to setup the sample platform for the PWB. The PWB fabrication equipment has an immersion lens, which means the lithography lens must be in close contact with the surface of the photonic chip, after which the photoresist fills the gap between the chip and lens. Since the designed photonic chip is smaller than the lens, the EWB wires will also be exposed under the lens if EWB is performed first. To avoid having the lens touching/damaging the electrical wire bonding, PWB is done first. We are aware that EWB’s ultrasonic power might shake the PWB during the EWB process, but the PWB is made of polymer, which is not rigid. We did not observe (under optical microscope) any obvious damage after the EWB. Moreover, the optical transmission response of the PWB chip did not change before and after the EWB.

Figure S11(a) shows the microscopic image of the photonic chip before the electrical wire bonding (EWB). The left-hand-side all-pass MRR (input MRR) serves as the input signal encoder, and the right-hand-side add-drop MRR filter (weight MRR) serves as the weight encoder, respectively. Figure S11(b) presents SEM images of the tip of the silicon taper with an oxide opening, which is used as the PWB interface to couple the light on and off the photonic chip. The chip was firstly sputtered with 5-nm-thick iridium (Leica EM ACE600) for SEM imaging. The roughness around the silicon taper indicates relatively high waveguide loss due to sidewall scattering. The trench around the waveguide taper is a side effect of the isotropic wet etching in the oxide opening process, which exposes the propagation mode to the sidewall due to a lower mode confinement. We believe the poor coupling efficiency should be attributed to these two factors, and better lithography processes may improve the performance.


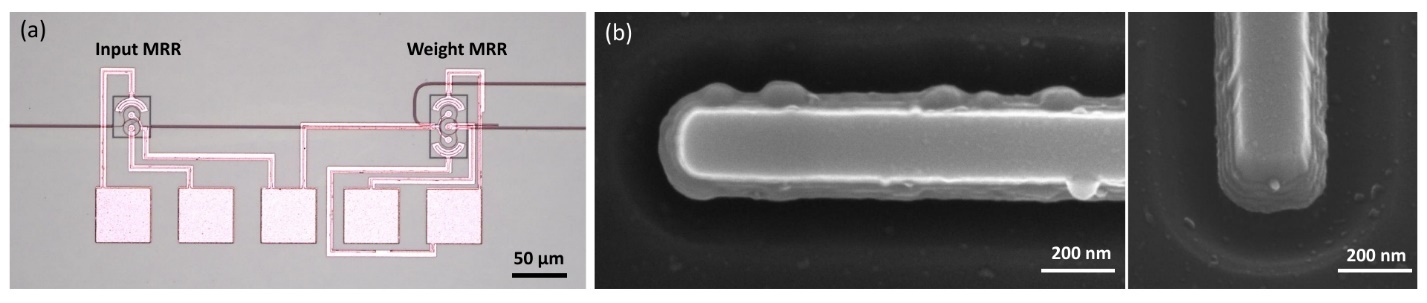


Figure S11. (a) Microscopic image of the photonic chip before co-packaging. (b) SEM images of the tip of the silicon taper for the PWB interface.

Figure S12(a) and S12(b) below shows the measurement setup for the co-packaged dot product photonic chip on the PCB. The PCB is attached using the heat conductive tape onto the TEC stage. Optical input and output fiber arrays on the PCB are connected to the tunable off-chip laser source (Optical in) and power meters (Optical out), respectively, to couple the light on and off the photonic chip. Electrical bias on ICPH and IRPH for dot product computation is applied by the source meter in the constant voltage mode through electrical I/Os wires.


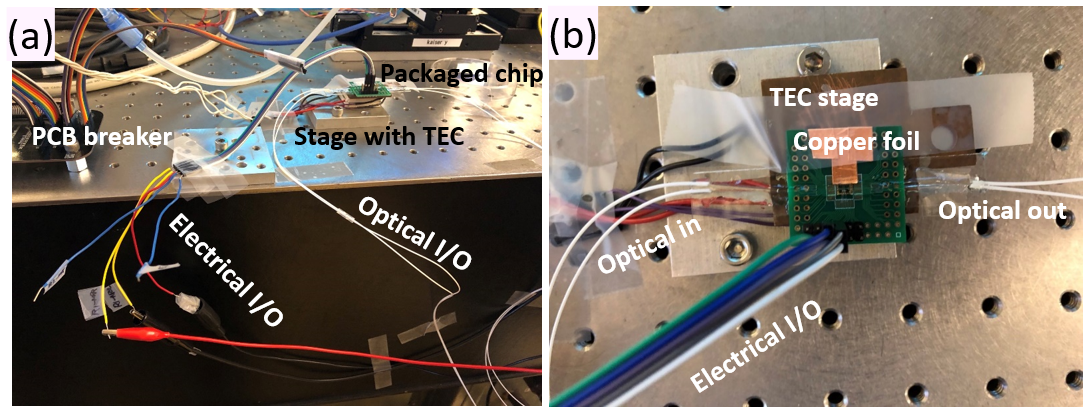


Figure S12. (a) The measurement setup for the co-packaged dot product photonic chip on the PCB. (b) Zoomed-in plot of the chip with electrical and optical I/Os.

**Reference**

1. Heebner, J., Grover, R. and Ibrahim, T., 2008. Optical microresonator theory (pp. 71-103). Springer New York.
2. Hai, M.S., Fard, M.M.P. and Liboiron-Ladouceur, O., 2016. A ring-based 25 Gb/s DAC-less PAM-4 modulator. IEEE Journal of Selected Topics in Quantum Electronics, 22(6), pp.123-130.
3. Popović, M., 2008. Theory and design of high-index-contrast microphotonic circuits (Doctoral dissertation, Massachusetts Institute of Technology).
4. Ansys-Lumerical. Lumerical. <http://www.lumerical.com/> (Accessed: 18 January 2022).
5. Tait, A.N., Wu, A.X., De Lima, T.F., Zhou, E., Shastri, B.J., Nahmias, M.A. and Prucnal, P.R., 2016. Microring weight banks. IEEE Journal of Selected Topics in Quantum Electronics, 22(6), pp.312-325.
6. Schwelb, O., 1998. Generalized analysis for a class of linear interferometric networks. I. Analysis. IEEE Transactions on Microwave Theory and Techniques, 46(10), pp.1399-1408.
7. Tait, A.N., 2018. Silicon photonic neural networks (Doctoral dissertation, Princeton University).
8. Bangari, V., Marquez, B.A., Miller, H., Tait, A.N., Nahmias, M.A., De Lima, T.F., Peng, H.T., Prucnal, P.R. and Shastri, B.J., 2019. Digital electronics and analog photonics for convolutional neural networks (DEAP-CNNs). IEEE Journal of Selected Topics in Quantum Electronics, 26(1), pp.1-13.
